# Methods for Generating and Evaluating Synthetic Longitudinal Patient Data: A Systematic Review

**DOI:** 10.1007/s41666-025-00223-7

**Published:** 2025-11-17

**Authors:** Katariina Perkonoja, Kari Auranen, Joni Virta

**Affiliations:** 1 Department of Mathematics and Statistics, University of Turku, Turku, Finland; 2 Department of Computing, University of Turku, Turku, Finland; 3 Department of Clinical Medicine, University of Turku, Turku, Finland

**Keywords:** Data sharing, Longitudinal patient data, Privacy-preserving data publishing, Statistical disclosure control, Synthetic data generation

## Abstract

The rapid growth in data availability has facilitated research and development, yet not all industries have benefited equally due to legal and privacy constraints. The healthcare sector faces significant challenges in utilizing patient data because of concerns about data security and confidentiality. To address this, various privacy-preserving methods, including synthetic data generation, have been proposed. Synthetic data replicate existing data as closely as possible, acting as a proxy for sensitive information. While patient data are often longitudinal, this aspect remains underrepresented in existing reviews of synthetic data generation in healthcare. This paper maps and describes methods for generating and evaluating synthetic longitudinal patient data in real-life settings through a systematic literature review, conducted following the PRISMA guidelines and incorporating data from five databases up to May 2024. Thirty-nine methods were identified, with four addressing all key challenges in longitudinal patient data generation: preserving temporal structure, heterogeneous variable types, missing values, and unbalanced data. Most studies assessed resemblance to real data, the majority evaluated utility, and just over half examined privacy. However, only a minority considered all three aspects together. While four methods addressed the key challenges in generating synthetic longitudinal patient data, none incorporated privacy-preserving mechanisms. Additionally, their effectiveness with small sample sizes remains unclear, raising concerns about their real-world applicability. The lack of standardized evaluation criteria further complicates comparison. Future research should focus on developing privacy-preserving methods, robust evaluation frameworks, and ensuring publicly accessible code. Clearer directives from data protection authorities are needed, as synthetic patient data availability lags behind method development.

**Supplementary Information:**

The online version contains supplementary material available at 10.1007/s41666-025-00223-7.

## Introduction

The recent surge in data volumes has greatly facilitated research, development, and innovation (RDI) activities. Yet, some sectors, particularly medicine, still face challenges in harnessing existing data sources due to stringent data protection regulations, such as the General Data Protection Regulation [1] or the Health Insurance Portability and Accountability Act [2]. Compliance with these policies leads to prolonged data processing times and, in certain cases, restricted access. For instance, while the national regulation in Finland permits using identifiable individual-level data for research, their use in development and innovation activities remain prohibited [3].

If patient data are deemed anonymous, they fall outside the rules of personal data protection, streamlining data access and sharing. Synthetic data generation (SDG), originally proposed by Rubin [4] in 1993, offers a promising approach to achieve such anonymity. The goal of SDG is to produce artificial data that resemble real-world observations, referred to as original or input data, while maintaining adequate utility and resemblance with the original. Here, resemblance refers to similarity between the synthetic and original data distributions, while utility pertains to the extent the analyses and predictions based on the synthesized data align with those from the original data.

Concerns regarding the sufficiency of mere random data generation for privacy preservation have prompted exploration of more effective privacy-preserving techniques in SDG [5, 6]. Beyond privacy, synthetic data (SD) help establish analytical environments and model testing in preparation for real data, while also enabling data augmentation for underrepresented observations (class imbalance). Consequently, SDG has gained prominence in enhancing data protection and facilitating RDI activities as well as educational purposes.

Medical data encompass various forms, with longitudinal patient data (LPD) being particularly valuable for their ability to provide detailed insights into patient trajectories over time. LPD are a form of tabular data that consist of at least one variable measured for each subject at two or more time points [7]. In addition to these time-varying repeated measurements, LPD often include static variables, such as patient demographics. The integration of static and time-varying variables in LPD enables a more comprehensive analysis of patient well-being. These insights extend beyond what can be derived from cross-sectional data that capture information only at a single time point [7].

For example, LPD are essential for monitoring disease progression [8], allowing clinicians to observe how conditions evolve over time and enabling timely interventions. They help in identifying early disease indicators [9], such as detecting subtle changes that signal the onset of various, potentially life threatening, conditions. By providing insights into individual responses to treatments, LPD facilitate the optimization of personalized treatment [10], improving patient outcomes. Additionally, LPD contribute to large-scale medical research [11, 12], enhancing understanding of disease patterns and treatment efficacies. The ability to develop predictive models using LPD also supports proactive healthcare planning by forecasting hospital readmissions or disease onset [12]. Beyond individual patient care, LPD identify population health trends and inform public health policies. Despite the immense potential of longitudinal patient data, access remains a significant challenge. Synthetic LPD can play a critical role in this context by facilitating different RDI activities while mitigating data privacy concerns.

Longitudinal patient data can be collected retrospectively, such as from electronic health records (EHRs), or prospectively, as in clinical trials and cohort studies. When repeated measurements are collected uniformly across subjects, LPD are considered *balanced*. Conversely, if the timing or frequency of measurements varies between subjects, LPD are called *unbalanced*. The question of unbalancedness is distinct from that of missing data; in an unbalanced dataset, the reasons for irregular observations are known and can be addressed based on this prior knowledge, whereas with missing data, both the cause and nature of the missing values must be examined [13]. Notably, missing data can occur in both balanced and unbalanced LPD structures.

**Fig. 1:**
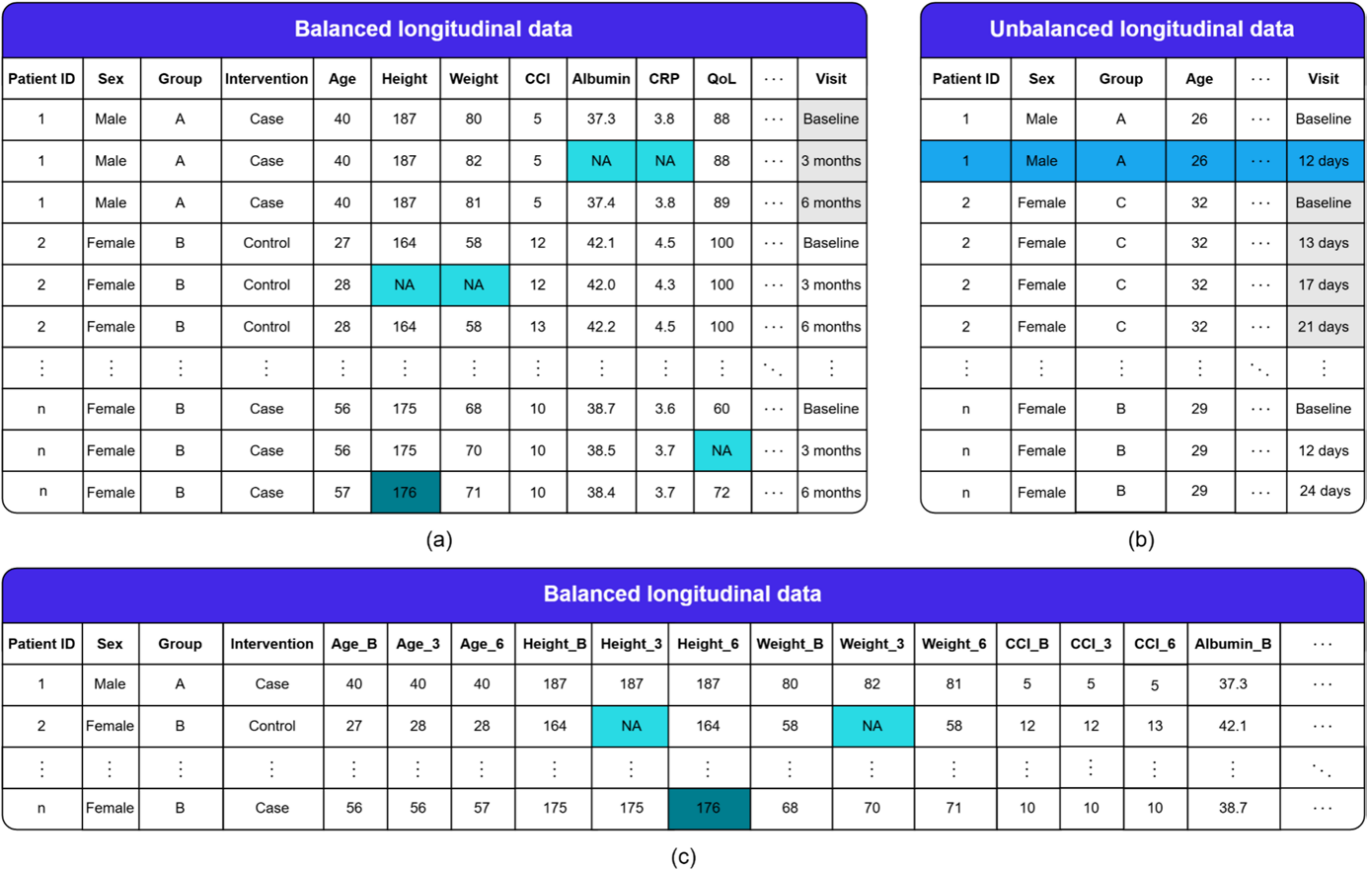
Illustration of key characteristics and different forms of longitudinal data. Subfigure (**a**) shows balanced (subjects have identical visit sequences), and subfigure (**b**) shows unbalanced (differing visit sequences) longitudinal data in long format (multiple rows per subject). The third subfigure (**c**) illustrates the same data as in (a) but in wide format (single row per subject). In this illustration, the focus could be in modeling the change in response variables such as quality of life (QoL) or albumin levels (reflecting disease progression) across intervention groups receiving distinct treatments, incorporating static variables (e.g., sex, group) and time-varying variables (e.g., age, height, weight) as covariates (features). The main difference to another common tabular data type, cross-sectional data, are precisely these repeatedly measured variables from the same subjects over time. The repeated measurements create a unique temporal structure that is essential for accurate analysis and synthetic data generation. Missing data (NA), measurement errors (176 in (a)), and dropouts (second row in (b)) are common issues encountered in longitudinal data and can complicate their analysis and synthetic data generation

The concept of unbalancedness in LPD should not be confused with class imbalance, which refers to the underrepresentation of specific categories or subgroups. However, significant unbalance in a dataset can contribute to class imbalance. Unbalanced LPD often arise in secondary data use due to individualized patient treatment, whereas prospective studies typically adhere to predefined measurement schedules, leading to more balanced data. Nonetheless, unbalance can occur in prospective studies, such as when cost constraints limit measurement frequency for certain participants. Figure 1 depicts the key characteristics of longitudinal patient data.

Longitudinal patient data are related but not equivalent to other time-dependent data types, of which time series and survival data are examples. Time series involve frequent, equally spaced repeated measurements, often with assumed dependencies between multiple series, and usually lack static variables [14]. Conversely, LPD consist of independent realizations of relatively short subject-specific series of observations. The key difference between longitudinal patient and survival data lies in their conceptualization of time and its role in data analysis. In LPD, time is treated as a fixed index, whereas in survival analysis, time-to-event is modeled as a random variable and is of primary interest [15].

### Rationale and Related Works

Both regular tabular, i.e., cross-sectional, and longitudinal SD generators encounter challenges when facing different variable types or missing data. However, using a regular tabular data generator for LPD can result in flawed generative models and manifest as logical inconsistencies, such as treating repeated within-subject measurements as independent entities, leading to eventual loss of temporal correlation [16], or inaccurately representing variability within or between subjects [7]. Unlike cross-sectional data, longitudinal patient data exhibit time-dependent relationships that must be preserved for synthetic data to be meaningful.

Cross-sectional data generators also struggle with the sparsity of unbalanced LPD and cannot separate “true missingness” from the unbalanced structure. While synthetic time-series generators can capture temporal relationships, they may inadequately replicate the underlying correlation structures due to their reliance on large volumes of equispaced repeated measurements. Furthermore, these generators often struggle to produce static variables in conjunction with the time series. This limitation is critical in medical applications, where static covariates (e.g., demographics) are integrated with time-dependent information (e.g., disease progression). Synthetic survival data generators focus on modeling a single time-to-event as a continuous variable, lacking potential in generating repeated measurements at discrete time points in LPD.

Consequently, additional research is warranted to identify appropriate techniques for generating synthetic longitudinal patient data that are reliable and of sufficient quality to be used in real-life settings. Such methods could be directly offered to data controllers to facilitate the use of patient data in different RDI and educational activities while safeguarding patient privacy. This systematic literature review aims to address this need and serves as a natural continuation to a number of previous reviews on SDG methods in healthcare [17–25] and in the broader context of tabular data [26].

While almost all preceding reviews recognize the prevalence of longitudinal patient data, they neither provide a formal definition nor examine LPD in sufficient depth. In all cases, the discussion of LPD with respect to SDG methods, their evaluation, and the assessment of generated synthetic data is overshadowed by other data types. Reviews [20, 22–26], for instance, treat LPD as merely one category among many, offering limited attention to characteristics that are distinctive of longitudinal data, such as balance or format (see Fig. 1). Even when inherent characteristics of LPD are mentioned, as in [18, 20, 23], the reviews do not examine these aspects in detail: they may note the characteristics but often do not assess whether SDG methods were evaluated for preserving these features or consider how such evaluations were performed. Discussion of applying statistical inference to the generated data is likewise frequently missing.

Several reviews also apply natural restrictions that further limit their scope in comparison to our work. Reviews [17, 18] concentrate exclusively on generative adversarial network-based approaches and [20] surveys generative AI methods, thereby limiting methodological breadth. Other reviews restrict their attention to specific perspectives, such as privacy-preserving techniques [21] or open-source implementations [24]. Finally, one non-review study [27] compiles a list of SDG methods for LPD as background for methodological research but does not examine the properties of these methods or their evaluation.

Hence, our systematic review addresses a significant gap in the literature by comprehensively surveying a critical type of medical data that has been acknowledged for their importance but never thoroughly reviewed in the context of SDG. Unlike earlier reviews, we frame our analysis entirely through the lens of LPD, highlighting often-overlooked aspects such as the evaluation of temporal dependencies, balance and the preservation of statistical inferences—topics that have not been adequately addressed in prior literature.

By concentrating solely on LPD, we avoid redundant discussions of cross-sectional and other medical data modalities that have already been covered in previous reviews. Furthermore, our adherence to the PRISMA guidelines [28] throughout the review distinguishes our work from previous studies, which often offered only partial compliance, typically limited to a flow diagram (see Section 3.1). This adherence not only demonstrates the feasibility of conducting a systematic methodological review according to established quality standards but also emphasizes the rigor of our approach.

**Fig. 2:**
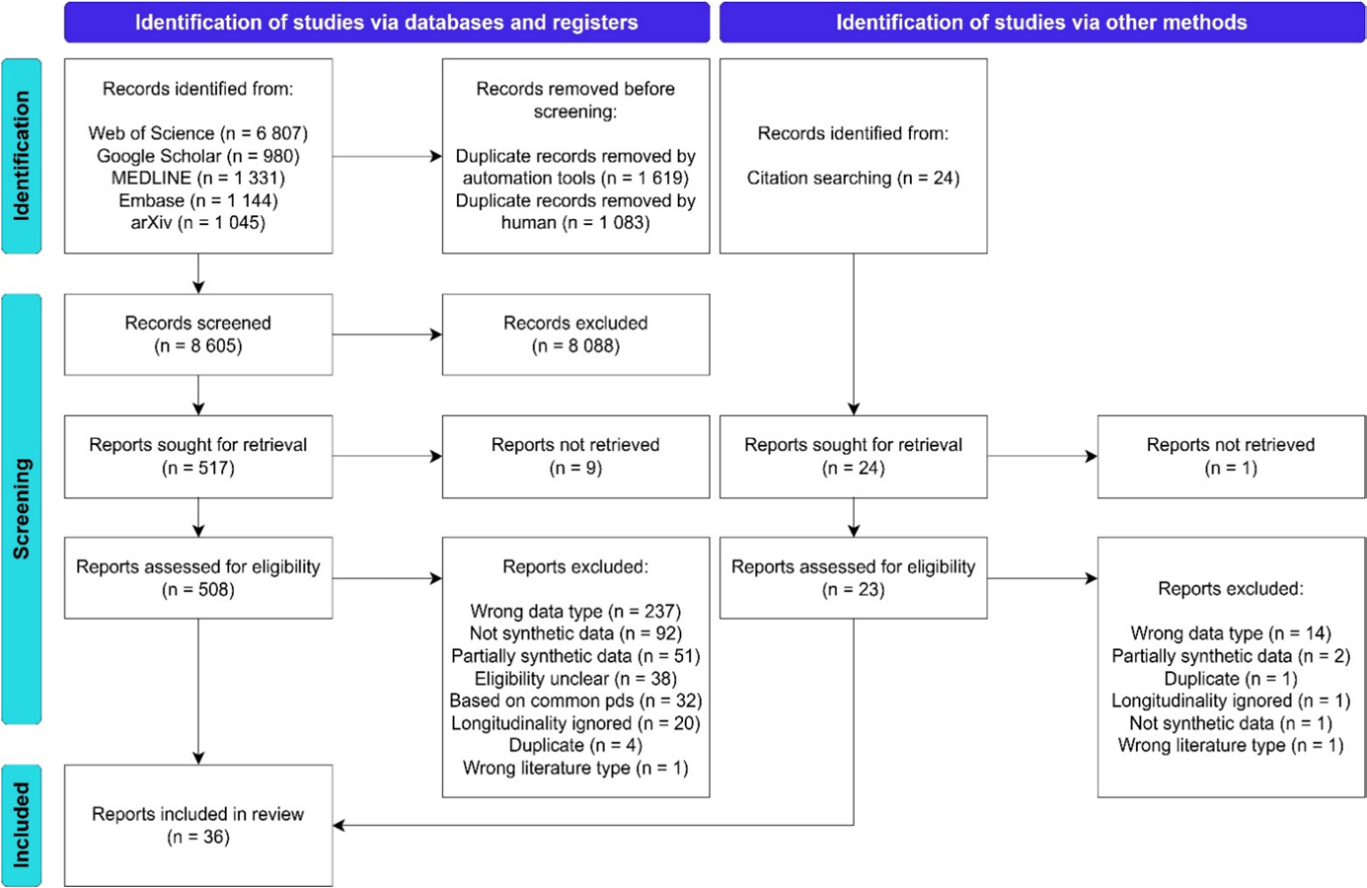
PRISMA flow diagram. The diagram illustrates the study selection process according to the PRISMA guidelines. Studies were excluded for several reasons: not generating synthetic data or relying on common probability distributions (Criterion 1); using the wrong data type or ignoring longitudinality (Criteria 2–3); using partially synthetic data (Criterion 4); or being the wrong literature type (Criterion 8). For 38 studies, eligibility could not be confirmed, and additional duplicates were removed. Examples of each exclusion category are provided in Supplementary material A.5

### Objectives

The primary objective of this systematic review is to map and describe existing methods of generating synthetic longitudinal patient data in real-life settings. The research questions under the primary objective are:

1. What methods are currently available for generating synthetic longitudinal patient data?
2. Do these methods address the key characteristics of longitudinal patient data, including temporal structure, balance, different variable types, and missing values?
3. How were these methods evaluated in terms of resemblance, utility, and privacy preservation?

The secondary objectives are to evaluate the comprehensiveness of reporting in the identified literature and to provide insights for method developers about areas requiring further research. Comparing the identified methods in practice is beyond the scope of this review but presents an intriguing prospect for future research.

The rest of the article is organized as follows: Sect. 2 outlines the review’s methodology, Sect. 3 presents the findings of individual studies and their synthesis. Section 4 concludes the article by offering general interpretations of the results, addressing limitations and discussing practical implications.

## Materials and Methods

This section outlines the methodological framework of the review, covering the eligibility criteria, search strategies, data items collected, and the approach used to present the results, with additional details available in the Supplementary materials.

### Eligibility Criteria

To address RQ1–3, we established specific selection criteria outlined in Table 1. We defined synthetic data as data generated using a randomized algorithm designed to mimic the original data while allowing the generation of an unlimited number of samples (Criterion 1). This definition is important, as distinguishing synthetic data from simulated data remains a challenge in literature.

**Table 1:** Eligibility criteria. The following criteria were applied to select relevant literature.

Number	Criterion	Description	Examples of exclusions
1	Includes synthetic data generation	Synthetic data are generated via a randomized algorithm using an existing dataset (i.e., original or input dataset) with the goal of closely mimicking the original data distribution and with the ability to generate unlimited number of synthetic samples	• Deterministic algorithm, e.g., rule-based • Algorithm based naively on common probability distributions • Data simulation, i.e., data are generated from theoretical models
2	Longitudinal data	The input and output dataset includes at least one repeatedly measured variable, and the authors address this longitudinal aspect	• Not longitudinal data • Longitudinal data altered so that the temporal structure is lost, e.g., through aggregation • Variables included “incidentally” without explicitly considering repeated measurements or temporal correlation
3	Data comparability	In case the input data are not patient data, variables in the original dataset should be comparable to those found generally in longitudinal patient data	• Data not comparable to longitudinal patient data
4	Data sharing capability	Method should support data sharing and generate fully synthetic data which is void of the original confidential data	• Study generates partially synthetic data
5	Privacy-preserving techniques	Consideration of privacy-preserving techniques in SDG is optional	
6	Non-open-source and commercial methods	Literature involving non-open-source and commercially licensed methods are included	
7	Language	English	• Language other than English
8	Publication types	Peer-reviewed journals and proceedings as well as pre-prints, books, book chapters and reviews	• Other than listed in the Description

For example, although biophysical simulation systems and pharmacokinetics models are commonly used in medicine, we classified them as simulated data since they are typically fine-tuned for specific scenarios using a particular dataset rather than being directly applicable to any dataset. In contrast, we emphasized that SDG methods should be implementable by users on their own data. Consequently, studies creating simulated data or generating SD from standard probability distributions (such as multivariate normal) after estimating their parameters were excluded due to their limited real-world applicability.

Given our focus on longitudinal patient data, compatibility with this data type was essential (Criteria 2–3). We included only tabular data with at least one repeatedly measured variable (≥ 2 measurements) and required the longitudinal structure to be preserved in the synthetic data. Studies were excluded if this longitudinal aspect was disregarded or lost, e.g., through aggregation or methodological constraints, or if they generated other data types, such as images, time series, survival or multimodal data.

With the overarching goal of facilitating data sharing in healthcare, we sought methods capable of generating fully synthetic individual-level data (Criterion 4). Consequently, publications generating partially synthetic data, either by generating only a subset of variables or specific types of observations to mitigate class imbalances, were excluded as the resulting data still contain confidential personal information. Privacy considerations are paramount in data sharing. However, due to the lack of official guidelines and consensus on additional privacy measures, we took a permissive approach, not mandating separate privacy-preserving mechanisms (Criterion 5).

Wanting to provide a wide range of methods for different RDI activities, we did not limit our scope to open-source methods and included non-peer-reviewed literature, while restricting our selection to English (Criteria 6–8).

### Information Sources

We searched EMBASE (1947/01/01—2024/05/22), MEDLINE (Ovid interface, 1946/01/01—2024/05/22), Web of Science (1900/01/01—2024/05/22) and Google Scholar (Publish or Perish software [29], first 1000 hits on 2021/06/18), and arXiv (open-source metadata [30] on 2024/05/22). These databases were chosen because they offer the best coverage [31] and the latest, unpublished methods.

### Search Strategy

Search algorithms were developed using topic (title, abstract, keywords) and text words related to synthetic longitudinal patient data. An example is given in Box 1. The search algorithms, detailed in Supplementary material A.1, utilized terms such as ‘synthetic’, ‘artificial’, ‘data’ and ‘record’, intended to cover all possible synonyms used to refer to synthetic data in the literature. Words such as ‘longitudinal’, ‘follow-up’, ‘trajectory’, ‘health’, ‘medical’ and ‘patient’ were used to capture works that deal with longitudinal patient data.

**Box 1** Search strategy example. The following is the search strategy used to identify relevant literature in the Web of Science Core Collection. TS refers to a topic search (including title, abstract, author keywords, and Keywords Plus), LA indicates the language of the publication, and DT specifies the document type. NEAR/3 is a proximity operator that retrieves records where the specified terms appear within three words of each other, in any order. To improve specificity, the search string was refined to exclude frequently encountered but clearly irrelevant topics such as synthetic aperture radar, artificial insemination, and synthetic seismograms.

**Figure:**
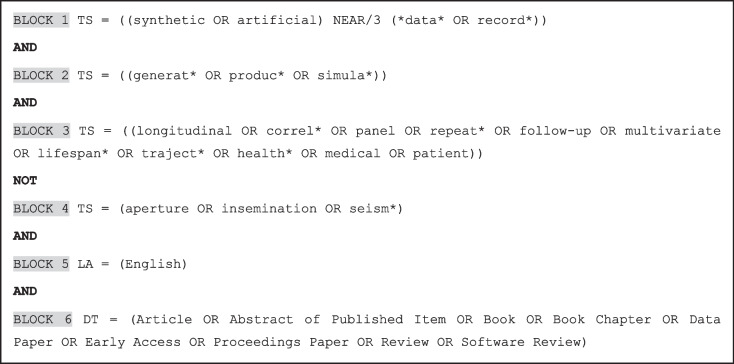

The initial algorithm was reviewed by an independent review team member using the PRESS standard [32]. To ensure timeliness, the search was conducted thrice in June 2021, November 2022 and May 2024. Additional records were identified through manual screening of the reference lists of eligible publications.

### Selection Process

After removing duplicates using EndNote Online [33], the results were uploaded to Rayyan [34] for literature screening. During the first two search iterations, the review authors (KP and JV) independently screened the titles and abstracts against the eligibility criteria using a specific screening chart (Supplementary material A.2.1) and leveraged Rayyan’s features to emphasize keywords indicative of inclusion or exclusion, streamlining the selection process. Any remaining duplicates were removed by KP.

Subsequently, full texts, referred to as studies or publications, were procured for records meeting the eligibility criteria or exhibiting any uncertainty in eligibility. Inaccessible publications were excluded. KP and JV independently assessed these full texts against the eligibility criteria using a specific full-text screening chart (Supplementary material A.2.2). Disagreements were resolved through discussion and, if necessary, a third-party arbitration (KA) was consulted. The reasons for exclusion were documented. The third search iteration followed the same principles, with KP conducting the screening and consulting JV for uncertain cases.

### Data Items and Collection

KP collected and managed data from the eligible publications using a structured form (Supplementary material A.3) within the REDCap electronic data capturing tool [35, 36]. In unclear situations, KP consulted the authors and their webpages to make sure that all relevant data were captured. For quality control, JV and KA conducted spot checks on the data collection process. Unavailable or unclear information was recorded as missing, and in cases of uncertainty, KP conferred with JV and KA.

### Risk of Bias and Reporting Quality Assessment

In adherence to the PRISMA guidelines [28], a systematic review is expected to consider and assess potential biases present in the included studies. Due to the absence of a specific evaluation framework in our context of a methodological review, we applied the existing guidelines from the Cochrane Handbook for Systematic Reviews of Interventions [37]. We considered events such as favoring data subsets for better results (selection bias), omission of results (reporting bias), and ambiguity in model performance assessment (performance bias). The detailed assessment framework is presented in Supplementary material A.4.1.

In the assessment of reporting quality, we adopted criteria inspired by the Grading of Recommendations, Assessment, Development and Evaluations framework [38], focusing on aspects such as the use of identical terminology or notations to denote disparate phenomena without clarification (inconsistency), a lack of precision or clarity in presenting information (imprecision), or conveying information in a less straightforward or explicit manner (indirectness). These factors pose a risk of misinterpretation, potentially leading to ambiguity or comprehension difficulties. Further details are provided in Supplementary material A.4.3.

KP assessed the risk of bias and the reporting quality, sharing the findings with JV and KA to obtain mutual agreement. Furthermore, KP collected data related to disclosed conflicts of interest and peer-review status.

### Methods to Address the Research Questions

To address RQ1, we compiled a concise overview encompassing all SDG methods that were predominantly applied in the eligible publications, subsequently referred to as the primary methods (Sect. 3.4). In addition, we recorded all SDG methods that were utilized for comparing and benchmarking against the primary methods, referred to as the reference methods.

To address RQ2, we constructed a comprehensive table (Sect. 3.4) outlining the characteristics and capabilities of the primary methods. To address RQ3, we constructed summary figures and tables outlining the utilized datasets and different evaluation approaches, which we grouped into three categories: resemblance, utility and privacy (Sect. 3.5).

To gain insight into the broader evaluation framework, we examined whether each evaluation task was conducted using a single or multiple independently generated synthetic datasets and whether the evaluation of the SD quality was conducted in relation to the original data or some other way.

In accordance with the secondary objectives, we categorized the research objectives of the eligible publications (Sect. 3.2), assessed bias and reporting quality (Sect. 3.3) and classified the primary methods based on their main operating principle (Sect. 3.4). Finally, we discussed our findings in relation to the existing literature to identify future research areas (Sect. 4).

## Results

This section presents the results of our review, including study selection, study characteristics, risk of bias and reporting quality, identified SDG methods for longitudinal patient data (RQ1–2), and evaluation approaches for synthetic data quality (RQ3). A broader discussion and interpretation of these findings is provided in Sect. 4.

### Study Selection

The search initially identified 11 307 publications. After removing 2 027 duplicates, 8 605 studies underwent title and abstract screening, leading to selection of 517 publications for full-text screening. Nine studies were unattainable, leaving 508 studies for evaluation against the eligibility criteria (Sect. 2.1). Altogether 33 of the 508 studies met our criteria and were included at this stage. To augment the search, KP examined all references in the 33 included studies. This process identified 24 potential publications, of which three were deemed eligible and incorporated in the review. Ultimately, 36 eligible studies were included in the review. Figure 2 illustrates the study selection process according to the PRISMA guidelines [28].

### Study Characteristics

The 36 included studies (Table 2) were published between 2016–2024, with 13 (36%) published in 2023 and nine (25%) in 2022. The predominant research objective in the studies was privacy-preserving data publishing (64%). Additionally, eight (22%) studies emphasized data publishing but did not employ privacy-preserving techniques or privacy evaluation. As per SCImago Journal & Country Rank [39], the most common publication fields were computer science (50%) and medicine (33%).

**Table 2:** Summary of the included publications. The table provides a summary of the 36 publications included in the systematic literature review. The publications are presented in descending order of publication year and sorted alphabetically by author name. The table includes information about the type of publication, the field based on SCImago Journal & Country Rank [39], and the objective of each publication as interpreted by the review authors. PPDP: privacy-preserving data publishing; (PP)DP: data publishing without considering data privacy.

Authors	Title	Outlet	Type	Field	Objective	Year
Belgodere et al. [40]	Auditing and Generating Synthetic Data with Controllable Trust Trade-offs	arXiv	Preprint	Multidisciplinary	Framework development	2024
Bun et al. [41]	Continual Release of Differentially Private Synthetic Data from Longitudinal Data Collections	Proc ACM Manag Data	Journal article	Computer science	PPDP	2024
Kühnel et al. [42]	Synthetic Data Generation for a Longitudinal Cohort Study – Evaluation, Method Extension and Reproduction of Published Data Analysis Results	Sci Rep	Journal article	Multidisciplinary	(PP)DP	2024
Pang et al. [43]	CEHR-GPT: Generating Electronic Health Records with Chronological Patient Timelines	arXiv	Preprint	Multidisciplinary	PPDP	2024
Theodorou et al. [44]	ConSequence: Synthesizing Logically Constrained Sequences for Electronic Health Record Generation	AAAI-24	Conference paper	Computer science	(PP)DP	2024
Das, Wang & Sun [45]	TWIN: Personalized Clinical Trial Digital Twin Generation	KDD ‘23	Conference paper	Computer science	PPDP	2023
El Kababji et al. [46]	Evaluating the Utility and Privacy of Synthetic Breast Cancer Clinical Trial Data Sets	JCO Clin Cancer Inform	Journal article	Biochemistry, genetics and molecular biology Medicine	PPDP	2023
Haleem et al. [47]	Deep-Learning-Driven Techniques for Real-Time Multimodal Health and Physical Data Synthesis	Electronics (Basel)	Journal article	Computer science Engineering	(PP)DP	2023
Hashemi et al. [48]	Time-series Anonymization of Tabular Health Data using Generative Adversarial Network	IJCNN	Conference paper	Computer science	PPDP	2023
Kuo et al. [49]	Generating Synthetic Clinical Data That Capture Class Imbalanced Distributions With Generative Adversarial Networks: Example Using Antiretroviral Therapy for HIV	JBI	Journal article	Computer science Medicine	PPDP	2023
Kuo et al. [50]	Synthetic Health-related Longitudinal Data with Mixed-type Variables Generated using Diffusion Models	NeurIPS 2023 Workshop	Conference paper	Computer science	PPDP	2023
Li et al. [51]	Generating Synthetic Mixed-Type Longitudinal Electronic Health Records for Artificial Intelligent Applications	NPJ Digit Med	Journal article	Computer science Health professions Medicine	PPDP	2023
Mosquera et al. [27]	A Method for Generating Synthetic Longitudinal Health Data	BMC Med Res Methodol	Journal article	Medicine	PPDP	2023
Nikolentzos et al. [52]	Synthetic Electronic Health Records Generated With Variational Graph Autoencoders	NPJ Digit Med	Journal article	Computer science Health professions Medicine	PPDP	2023
Sood et al. [53]	Bayesian Network Modeling of Risk and Prodromal Markers of Parkinson’s Disease	PLoS One	Journal article	Multidisciplinary	PPDP	2023
Sun, Lin & Yan [54]	Collaborative Synthesis of Patient Records through Multi-Visit Health State Inference	arXiv	Preprint	Multidisciplinary	PPDP	2023
Theodorou, Xiao & Sun [55]	Synthesize High-Dimensional Longitudinal Electronic Health Records via Hierarchical Autoregressive Language Model	Nat Commun	Journal article	Biochemistry, genetics and molecular biology Chemistry Physics and Astronomy	PPDP	2023
Yoon et al. [56]	EHR-Safe: Generating High-Fidelity and Privacy-Preserving Synthetic Electronic Health Records	NPJ Digit Med	Journal article	Computer science Health professions Medicine	PPDP	2023
Bhanot et al. [57]	Investigating Synthetic Medical Time-Series Resemblance	Neurocomputing	Journal article	Computer science Neuroscience	Resemblance quantification	2022
Kuo et al. [58]	The Health Gym: Synthetic Health-Related Datasets for the Development of Reinforcement Learning Algorithms	Sci Data	Journal article	Computer science Decision science Mathematics Social Sciences	PPDP	2022
Lu et al. [59]	Multi-Label Clinical Time-Series Generation via Conditional GAN	arXiv	Preprint	Multidisciplinary	(PP)DP	2022
Shi et al. [60]	Generating High-Fidelity Privacy-Conscious Synthetic Patient Data for Causal Effect Estimation With Multiple Treatments	Front Artif Intell	Journal article	Computer science	PPDP	2022
Wang & Sun [61]	PromptEHR: Conditional Electronic Healthcare Records Generation with Prompt Learning	Proc Conf Empir Methods Nat Lang Process	Conference paper	Computer science	PPDP	2022
Wang et al. [62]	Using an Optimized Generative Model to Infer the Progression of Complications in Type 2 Diabetes Patients	BMC Med Inform Decis Mak	Journal article	Computer science Medicine	Modeling	2022
Wendland et al. [63]	Generation of Realistic Synthetic Data Using Multimodal Neural Ordinary Differential Equations	NPJ Digit Med	Journal article	Computer science Health professions Medicine	(PP)DP	2022
Yu, He & Raghunathan [64]	A Semiparametric Multiple Imputation Approach to Fully Synthetic Data for Complex Surveys	J Surv Stat Methodol	Journal article	Decision sciences Mathematics Social sciences	PPDP	2022
Zhang, Yan & Malin [16]	Keeping Synthetic Patients on Track: Feedback Mechanisms to Mitigate Performance Drift in Longitudinal Health Data Simulation	JAMIA	Journal article	Medicine	Framework development	2022
Biswal et al. [65]	EVA: Generating Longitudinal Electronic Health Records Using Conditional Variational Autoencoders	PMLR Machine Learning for Healthcare	Conference paper	Computer science and technology Computing Data processing	PPDP	2021
Zhang et al. [66]	SynTEG: A Framework for Temporal Structured Electronic Health Data Simulation	JAMIA	Journal article	Medicine	PPDP	2021
Gootjes-Dreesbach et al. [67]	Variational Autoencoder Modular Bayesian Networks for Simulation of Heterogeneous Clinical Study Data	Front Big Data	Journal article	Computer science	PPDP	2020
Sood et al. [68]	Realistic Simulation of Virtual Multi-Scale, Multi-Modal Patient Trajectories Using Bayesian Networks and Sparse Auto-Encoders	Sci Rep	Journal article	Multidisciplinary	(PP)DP	2020
Beaulieu-Jones et al. [69]	Privacy-Preserving Generative Deep Neural Networks Support Clinical Data Sharing	Circ Cardiovasc Qual Outcomes	Journal article	Medicine	PPDP	2019
Fisher et al. [70]	Machine Learning for Comprehensive Forecasting of Alzheimer’s Disease Progression	Sci Rep	Journal article	Multidisciplinary	Modeling	2019
Barrientos et al. [71]	Providing Access to Confidential Research Data Through Synthesis and Verification: An Application to Data on Employees of the U.S. Federal Government	Ann Appl Stat	Journal article	Decision sciences Mathematics	PPDP	2018
Walonoski et al. [72]	Synthea: An Approach, Method, and Software Mechanism for Generating Synthetic Patients and the Synthetic Electronic Health Care Record	JAMIA	Journal article	Medicine	(PP)DP	2018
Raab, Nowok & Dibben [73]	Practical Data Synthesis for Large Samples	J Priv Confid	Journal article	Computer science Mathematics	(PP)DP	2018

### Risk of Bias and Reporting Quality

The included studies showed no indication of selection bias. However, 11 studies (28%) had potential risk of performance bias due to inadequate transparency in their evaluation descriptions, particularly regarding model training of the reference methods, or due to the unavailability of source code needed to verify their evaluation processes.

A risk of reporting bias was observed in 17 publications (44%). While the specific issues varied, the primary reason was the absence or partial presentation of results that were described in the methodology but not reported in the main results or supplemental materials. Further details are available in Supplementary material A.4.2.

Inconsistency of reporting was observed in one study, imprecision in six (15%), and evidence of indirect reporting in seven studies (18%). These findings related to presentation of p-values, sample size, included variables, evaluation metrics and privacy budget with differing degrees of precision or notation. Further details are given in Supplementary material A.4.3.

In five studies (13%) the documentation of the training processes was only partially provided, and 15 publications (38%) lacked them altogether. Among the included publications, only four (11%) were not peer reviewed, consisting exclusively of arXiv preprints. Conflict of interest was declared in eight (21%) publications, whereas this information was absent in 14 (36%) publications.

### SDG Methods for Longitudinal Patient Data

Given the diversity of longitudinal patient datasets reported in the literature, we grouped them into three categories. Methods generating both static and time-varying variables were classified as producing “standard data”. Those generating only time-varying variables without static variables were classified as producing “trajectory data” (e.g., diagnostic codes, procedure codes, and prescriptions per visit). Finally, methods generating a single repeatedly measured variable, such as a disease code, were classified as producing “sequence data”.

**Fig. 3:**
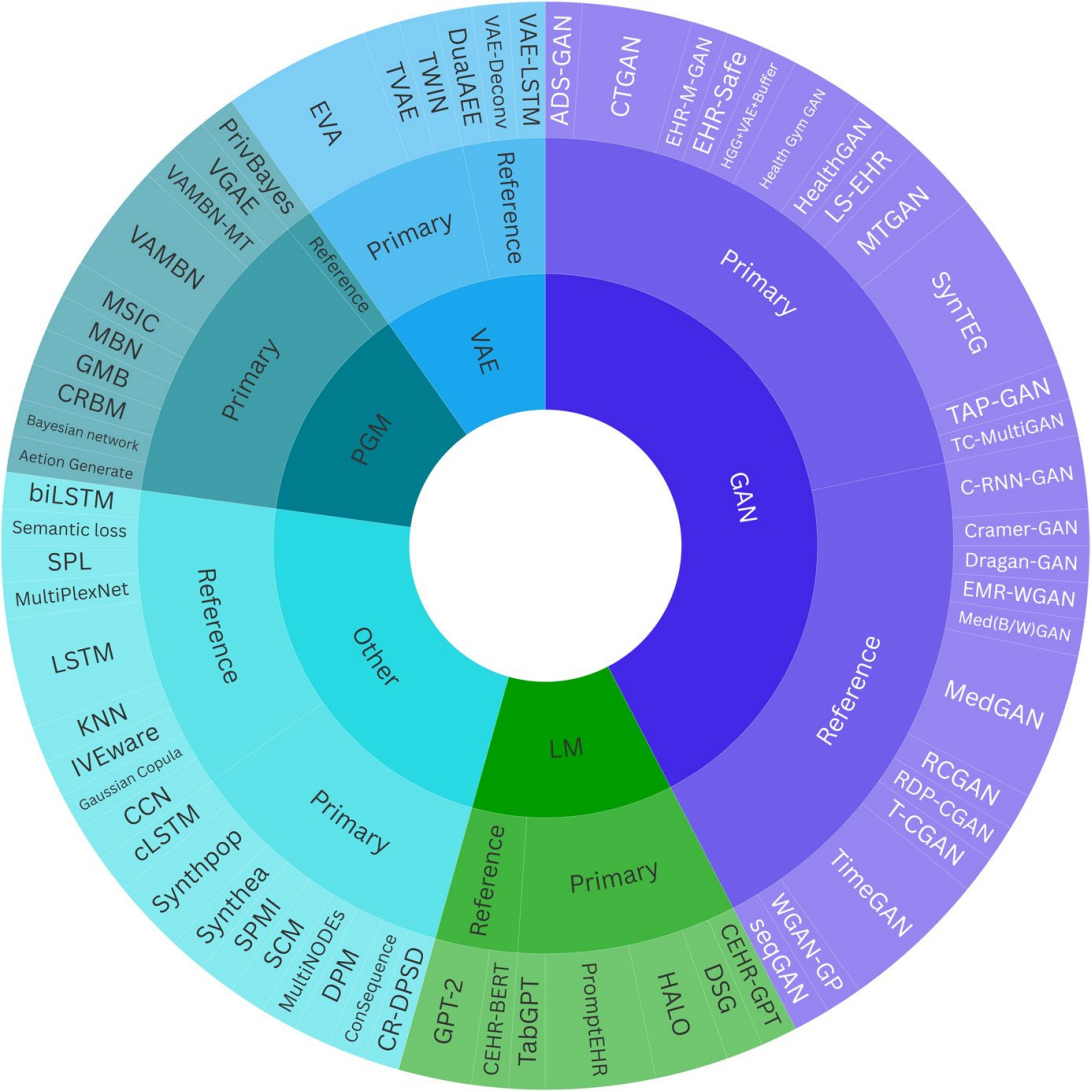
Categorization of the identified SDG methods. The 39 primary methods and 27 reference methods identified were classified into five distinct groups according to their main operating principle: generative adversarial networks (GANs), language models (LMs), variational autoencoders (VAEs), probabilistic graphical models (PGMs), and others. The width of each sector represents the frequency of method use across the 36 publications reviewed

In total, we identified 66 SDG methods, comprising 39 primary and 27 reference methods (Supplementary material A.6). Most primary methods (67%) were designed to generate standard LPD, while eight methods (21%) generated sequence data and six (15%) generated trajectories. Figure 3 illustrates a categorization of all identified methods according to their key operating principles while Table 3 details the key characteristics of the primary methods.

**Table 3:** Summary of the 39 identified primary synthetic longitudinal patient data generation methods. The table summarizes each method, its key operating principle, the type of synthetic longitudinal patient data generated, its ability to generate unbalanced and missing data structures, as well as categorical and continuous variables (✓ Yes, ✗ No,? Unclear). Additionally, the table specifies whether expert knowledge is required, the availability of source code and pseudocode (referenced in parentheses if not from the listed publication), and the programming language used. The final column lists all identified publications where the method was applied. Ideally, the optimal method would have a ✓ in every column.

Method	Class	Data type	Unbalanced data	Missing data	Categorical variables	Numerical variables	No expert knowledge	Privacy mechanism	Source/ pseudocode	Prog. language	Applied in
AC-GAN	GAN	Standard	?	✗	✓	✓	✓	✓	✓/✗	Python	[69]
ADS-GAN	GAN	Standard	?	✗^*^	✓	✓	✓	✓	✓[79]/✗	Python	[60]
CTGAN	GAN	Standard	✓	✗	✓	✓	✓	✗	✓ [79]/✗	Python	[46, 47, 59]
EHR-Safe	GAN	Standard	✓	✓	✓	✓	✓	✗	✗/✓	Python	[56]
Health Gym GAN	GAN	Standard	?	✗	✓	✓	✓	✗	✓/✗	Python	[49, 58]
HealthGAN	GAN	Standard	?	✗	✓	✓	✓	✗	✓ [80]/✗	Python	[57]
HGG-VAE-Buffer	GAN	Standard	✗	✗	✓	✓	✓	✗	✗/✓	Python	[49]
TC-MultiGAN	GAN	Standard	✗	✗^*^	✓	✓	✓	✗	✗/✗	Python	[47]
CEHR-GPT	LM	Standard	✓	?	✓	✗	✓	✗	✗/✗	?	[43]
DSG	LM	Standard	✓	✓	✓	✓	✓	✗	✗/✗	Python	[47]
TabGPT	LM	Standard	?	?	✓	✓	✓	✓	✓ [81]/✗	Python	[40]
HALO	LM	Standard Sequence	✓	✓	✓	✓	✓	✗	✓/✗	Python	[44, 55]
Aetion Generate^†^	PGM	Standard	✗	?	✓	✓	✓	?	✗/✓ [82]	Python + R	[46]
Bayesian network	PGM	Standard	?	✗^*^	✓	✓	✗	✗	✗/✗	R	[53]
MBN	PGM	Standard	✗	✗^*^	✓	✓	✗	✗	✓^**^/✗	R	[68]
VAMBN	PGM	Standard	?	✗^*^	✓	✓	✗	✗	✓/✗	Python + R	[42, 63, 67]
VAMBN-MT	PGM	Standard	?	✗^*^	✓	✓	✗	✗	✓/✗	Python + R	[42]
TVAE	VAE	Standard	✓	✗	✓	✓	✓	✗	✓ [79]/✗	Python	[46]
cLSTM	Other	Standard	✓	✓	✓	✓	✓	✗	✓^**^/✗	Python	[27]
CRBM	Other	Standard	✗	✗^*^	✓	✓	✓	✗	✗/✗	?	[70]
DPM	Other	Standard	?	✗	✓	✓	✓	✗	✗/✗	Python	[50]
MultiNODEs	Other	Standard	?	✗^*^	✓	✓	✓	✗	✓/✗	Python + R	[63]
SCM	Other	Standard	?	✓	✓	✓	✗	✓	✓/✗	R	[71]
SPMI	Other	Standard	?	✗^*^	✓	✓	✓	✗	✓^**^/✗	R	[64]
Synthea	Other	Standard	?	✗	✓	✓	✗	✗	✓/✗	Java	[72]
Synthpop	Other	Standard	?	✓	✓	✓	✗	✗	✓/✗	R	[64, 73]
EHR-M-GAN	GAN	Trajectory	?	✗	✓	✓	✓	✓	✓/✓	Python	[51]
TAP-GAN	GAN	Trajectory	?	✗	✓	✓	✓	✓	✗/✗	Python	[48]
PromptEHR	LM	Trajectory	?	?	✓	✗	✓	✗	✓/✗	Python	[45, 54, 61]
MSIC	PGM	Trajectory	?	?	✓	✗	✓	✗	✗/✓	?	[54]
VGAE	PGM	Trajectory	?	?	✓	?	?	✓	✓^**^/✗	?	[52]
TWIN	VAE	Trajectory	✗	?	✓	✗	✓	✗	✓/✗	Python	[45]
LS-EHR	GAN	Sequence	?	✗	✓	✗	✓	✗	✗/✗	?	[16]
MTGAN	GAN	Sequence	?	✗	✓	✗	✓	✗	✓/✓	Python	[54, 59]
SynTEG	GAN	Sequence	?	✗	✓	✗	✓	✗	✗/✗	?	[45, 51, 55, 61, 66]
GMB	PGM	Sequence	✓	✗	✓	✗	✗	✗	✗/✓ [83]	Python	[62]
EVA	VAE	Sequence	✓	✗	✓	✗	✓	✗	✗/✗	?	[45, 54, 55, 65]
ConSequence	Other	Sequence	✓	✓	✓	✗	✗	✗	✓/✗	Python	[44]
CR-DPSD	Other	Sequence	✗	✗	✓	✗	✗	✓	✗/✓	?	[41]

^*^ Imputed prior SDG; ^**^ Upon request.

All primary methods permitted user customization or training for specific datasets, except for Synthea. Synthea is a widely recognized tool for generating synthetic patient data and relies on a pre-built disease module generator but also supports user-created modules [74], which provides functionality comparable to dataset-driven methods, prompting us to include Synthea among the identified methods. Of the included methods, 10 (26%) required expert knowledge, meaning they demand detailed data-specific information for their effective use.

Different method categories employed varying approaches to model the temporal structure of LPD. Generative adversarial networks (GANs) utilized convolutional layers and recurrent neural networks (RNNs), such as gated recurrent units or long short-term memory (LSTM) networks, which are adept at handling sequential data [75–78]. Probabilistic graphical models (PGMs) captured temporal structure by constraining node interactions, ensuring future values being modeled based on preceding values and relevant covariates. Variational autoencoders (VAEs) utilized autoregressive techniques, feeding data sequentially while latent variables accounted for patient-specific heterogeneity. Language models (LMs) modeled temporal data in a manner similar to sequential text generation utilizing transformer architectures and self-attention mechanisms [71]. Other methods employed a variety of techniques, but the most prevalent approaches were (a) the Markov assumption, where the present depends only on the previous time point, and (b) ordered generation of each subsequent variable, including the non-temporal variables, conditionally on all previously generated variables (instead of just the most recent one).

Information about the method’s ability to generate unbalanced data was available for 17 primary methods (44%), of which 10 could perform this task, though three were restricted to sequence data. All ten methods utilized sequence-to-sequence techniques, which support varying lengths of input and output data [84], to create the unbalanced structure.

Seven primary methods (18%) could generate missing observations. However, for four of these, it is unclear whether the missingness was primarily due to the unbalanced structure or if the method also modeled the missing value pattern. EHR-Safe [56] was the only method confirmed to generate unbalanced data while simultaneously modeling missing value patterns using a masking technique. Additionally, nine methods (23%) required imputation of missing values in the original data before generating SD.

While all primary methods could generate categorical variables, many required one-hot encoding. Out of the 39 primary methods, 27 (69%) were able to generate numerical variables; however, some of these methods required discretization of numerical data before SDG. Privacy mechanisms were incorporated in eight methods (21%), including four utilizing differential privacy (DP) [85, 86], two incorporating penalties in the optimization algorithm, and two applying other noise perturbation.

Source codes were available for 23 primary methods (59%), with five (13%) available in another publication and four upon request. Pseudocodes were given for eight methods (21%), two of which were provided in a cited publication. Both the source and pseudocodes were inaccessible for 10 methods (26%). Python was the most common programming language (64%), followed by R (23%), and the programming language for eight primary methods (21%) was unverifiable. System requirements were detailed in 11 studies (28%).

### Evaluation Approaches for Synthetic Data Quality

Most studies (69%) generated a single synthetic dataset, while 25% produced a small number (< 50) of datasets, and 2 studies (6%) generated a larger quantity (≥ 50). Twelve studies (33%) explored the impact of adjusting hyperparameters or altering the method’s structural configuration on the quality of synthetic data, and 16 studies (44%) compared their primary method to reference methods (see Supplementary material A.6). The MIMIC-III dataset [87] was the most frequently used, appearing in 11 studies (31%). Two studies augmented their assessment with simulated data. The complete list of datasets available in Supplementary material A.7.

Almost all studies (94%) compared SD to the original data in at least one of the following areas: resemblance, utility, or privacy, with [40] performing these comparisons in an embedding space. The remaining two studies pursued alternative approaches: [72] compared prevalence statistics in SD against empirical population data, and [62] focused solely on describing the characteristics of the SD. The subsequent subsections detail the approaches used to evaluate resemblance, utility, and privacy.

#### Resemblance

We distinguished four domains within resemblance: univariate distributions, bivariate distributions, multivariate distributions, and temporal structure. Approaches in the univariate domain assessed resemblance by comparing the values $s\left(X\right)$ and $s\left({X}^{*}\right)$ of a given summary statistic $s\left(\bullet \right)$, where $X$ and ${X}^{*}$ are the marginal distributions of a single variable over all subjects and all time points in the original and synthetic data, respectively. Approaches in bivariate and multivariate domains followed the same principle but considered the marginal distributions of a pair or a collection of variables, respectively. Finally, approaches in the temporal structure domain explicitly accounted for measurement time and compared the joint distribution ${X}_{{t}_{1}},{X}_{{t}_{2}},\dots$ of the original data across multiple time points ${t}_{1},{t}_{2},\dots$ with its synthetic counterpart through some summary statistic $s\left(\bullet \right)$.

For each of these domains, the evaluation approaches were further divided into four paradigms: qualitative (e.g., visual inspection through figures), quantitative (based on summary statistics or other numerical measures), model-based (fitting models such as principal component analysis or factor analysis to compare structural similarity), and statistical test-based. Approaches that did not fall into any of these paradigms were grouped under the category “Other.”

**Fig. 4:**
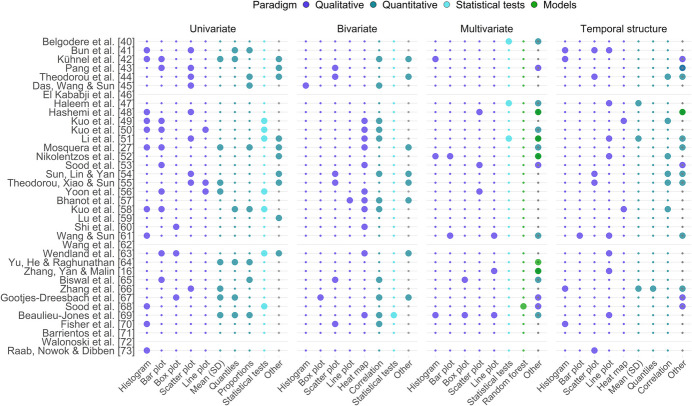
Approaches used to evaluate synthetic and original data resemblance. The x-axis lists approaches used across studies for assessing the resemblance between the synthetic and original data; larger circles indicate use, and gray lines mark studies that did not assess resemblance. Resemblance is evaluated across four domains: univariate, bivariate, and multivariate distributions as well as temporal structure, using approaches classified into qualitative, quantitative, statistical test-based, and model-based paradigms.: A single study may employ multiple assessments within the same dimension. The “Histogram” approach also includes density plots, and the “Box plot” approach encompasses violin plots. Statistical tests, models and approaches labeled as “Other” are discussed in the main text. SD: standard deviation

Thirty-three studies (92%) assessed resemblance between the synthetic and original data (Fig. 4). Among these studies, seven (21%) evaluated all four domains. Similarly, 14 studies (42%) evaluated three domains, eight studies evaluated two domains, and three studies (9%) evaluated one domain. Univariate resemblance was assessed in 28 of the 33 studies (85%), 20 studies (61%) examined bivariate resemblance, 18 studies (55%) evaluated multivariate resemblance, and 23 studies (70%) investigated temporal resemblance.

In the univariate domain (Fig. 4, panel 1), qualitative paradigms were applied in 24 studies (73%), histograms being the most prevalent approach. Quantitative paradigms were employed in 19 studies (58%), primarily focusing on evaluating means, standard deviations, quantiles, and proportions. “Other” approaches to quantitative comparisons included, e.g., root mean square error (RMSE) and Jensen-Shannon divergence. Statistical tests were employed in seven studies (21%), including Mann–Whitney U, t-, or Kolmogorov–Smirnov (KS) tests for continuous variables, and Chi-squared homogeneity test for categorical variables.

In the bivariate domain (Fig. 4, panel 2), quantitative and qualitative paradigms were used in 19 (58%) and 16 (48%) studies, respectively. The most common approaches were comparing correlations and the corresponding heatmaps. “Other” quantitative approaches included calculating norms and errors for correlation and transition matrices. One study assessed the statistical significance of correlation coefficients. Evaluations of categorical variables, particularly their comparison with continuous variables, were less frequent.

In the multivariate domain (Fig. 4, panel 3), qualitative paradigms were employed in 12 studies (36%), and primarily utilized visualizations of dimensionality reduction techniques, such as principal component analysis and t-distributed stochastic neighbor embedding. Quantitative paradigms, implemented in 11 studies (33%), included metrics such as clinician-assigned scores and results of model-based evaluations, e.g., area under the curve (AUC) and classification accuracy. In six studies (18%), models like random forests, support vector machines, LSTMs, and “Other” models (Jensen-Shannon divergence-based discriminators) were trained to distinguish real patients from synthetic ones based on a set of variables, with performance close to random guessing indicating high resemblance. Factor analysis, also included under “Other” models, was used separately to explore structural similarities between datasets. Additionally, three studies (9%) employed statistical tests, including the KS-test, maximum mean discrepancy across multiple variables, and t-test to infer discriminator performance. Additional details on the identified model-based paradigms can be found in Supplementary Material A.8.

Temporal resemblance assessment (Fig. 4, panel 4) between synthetic and original variables was qualitatively assessed in 21 studies (64%), primarily through visualizing mean values over time using line plots or consecutive measurements with scatter plots. “Other” qualitative approaches involved visualizing underlying network structures and using tile plots. Quantitative paradigms were used in 13 studies (39%), including the calculation of means, standard deviations for visit intervals, and autocorrelation functions. “Other” quantitative approaches included calculating the RMSE of autocorrelation functions, a singular value-based latent temporal statistic, bigram sequences, and metrics assessing the loss of temporal information, trends, and cycles. One study applied a model-based approach, predicting the next value based on previous ones using an RNN.

#### Utility

For utility we distinguished two domains: statistical inference and prediction performance (including classification tasks). Approaches that imposed a probabilistic model on the data and compared the estimates or results of hypothesis tests concerning a subset of its parameters across the original and synthetic data were considered statistical inference. In contrast, prediction performance involved using a model $f\left(\bullet \right)$ to predict the value of a variable $y$ from the remaining variables $X$, with prediction accuracies compared across original and synthetic data. To further classify the utility approaches within each domain, we applied the same four paradigms used for resemblance.

**Fig. 5:**
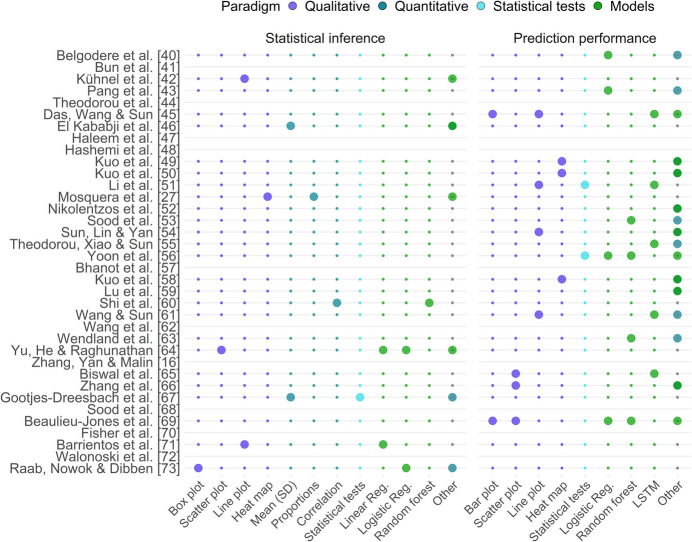
Approaches used to evaluate synthetic data utility in comparison to the original data. The x-axis lists approaches used across studies for assessing the utility between the synthetic and original data; larger circles indicate use, and gray lines mark studies that did not assess utility. Utility is evaluated across two domains: similarity of statistical inference and prediction performance, including classification., using approaches classified into qualitative, quantitative, statistical test-based, and model-based paradigms. A single study may employ multiple assessments within the same dimension. Statistical tests and approaches labeled as “Other” are discussed in the main text. SD: standard deviation; Reg.: regression; LSTM: long short-term memory

Twenty-six studies (72%) assessed utility (Fig. 5) through clinically meaningful questions, ranging from simple predictions of a single variable, such as the presence of a specific condition or disease, to replicating the outcomes of real clinical trials. Eight of these 26 studies (31%) evaluated the similarity of statistical inferences between the synthetic and original data (Fig. 5, panel 1). Seven of the eight studies employed both qualitative and model-based approaches, while one focused on comparing the results of the Mann–Whitney U test. Qualitative paradigms were primarily used for visualizing model parameter estimates and confidence interval overlap. Additionally, “Other” quantitative approaches included evaluating ratios of standard errors and effect size. The models utilized included linear regression (using change in scores as a response), logistic regression (without accounting for longitudinality), causal effect models (results compared using different goodness-of-fit and correlation metrics), and two methods commonly used for longitudinal data: mixed-effects models and generalized estimating equations [88]. Additional details on the identified model-based paradigms can be found in Supplementary Material A.8.

Eighteen studies (69%) assessed predictive performance (Fig. 5, panel 2), all using model-based paradigms. These studies trained models with both synthetic and original data, comparing performance on a test set. The models employed included logistic regression, random forest, LSTM, and other methods such as support vector machines, RNN, multilayer perceptron, and batch-constrained Q-learning. In most prediction or classification tasks, the output variable was binary, typically representing the presence or absence of a specific disease condition, while the input variables included repeated measurements. Depending on the modeling approach, the data had to be structured in either long or wide format to appropriately account for longitudinality (Fig. 1). Ten studies used qualitative approaches to visualize model performance. Model performance metrics—such as accuracy, AUC, recall, and F_1_-score—were classified under “Other” quantitative approaches and compared in 13 studies. Two studies used t-tests to statistically assess differences in prediction performance between models.

#### Privacy

Privacy concerns, distinct from the privacy preservation techniques employed within the SDG method itself, were addressed in 19 studies (53%), as outlined in Table 4. These studies utilized a range of threat models to address various privacy concerns, with six studies examining two separate aspects.

**Table 4:** Approaches for evaluating privacy in synthetic data. The table summarizes studies that assessed various disclosure risks. Attribute disclosure occurs when sensitive or private information about an individual can be inferred from synthetic data. Identity disclosure happens when an individual’s identity is linked to their data. Membership disclosure involves the identification of an individual’s participation in the original dataset. Note that membership and identity disclosure are closely related concepts (theoretically, the latter implies the former) and therefore not always evaluated separately.

Study	Attribute disclosure	Identity disclosure	Membership disclosure
Belgodere et al. [40]			✓
Pang et al. [43]	✓		✓
Das, Wang & Sun [45]	✓		✓
El Kababji et al. [46]	✓		✓
Hashemi et al. [48]		✓	
Kuo et al. [49]		✓	
Kuo et al. [50]		✓	
Li et al. [51]			✓
Mosquera et al. [27]	✓		
Nikolentzos et al. [52]		✓	
Sun, Lin & Yan [54]	✓		✓
Theodorou, Xiao & Sun [55]	✓		✓
Yoon et al. [56]			✓
Kuo et al.[58]		✓	
Shi et al. [60]		✓	
Wang & Sun [61]	✓		✓
Biswal et al. [65]			✓
Zhang et al. [66]	✓		✓
Barrientos et al. [71]	✓		

Most of these studies (58%) focused on membership disclosure where an adversary, given some target record $x$, tries to infer whether $x$ is in the real data $D$ using the synthetic dataset ${D}_{syn}$. Two common approaches were distance-based and classifier-based attacks. In distance-based attacks, the similarity between synthetic and training data was compared to that between synthetic data and a hold-out test set using a predefined distance metric $d\left(\bullet ,\bullet \right)$. Specifically, if $d\left({x}^{*},{D}_{train}\right)\approx d\left({x}^{*},{D}_{test}\right)$, for a synthetic record ${x}^{*}$, then the disclosure risk was considered low. In other words, the synthetic record resembled the real training data no more than it did unrelated test data, making it unlikely to reveal whether any individual was part of the original dataset. In classifier-based attacks, a model $f\left(\bullet \right)$ was trained to distinguish between real and synthetic samples by minimizing a loss function $L\left(f\left(x\right),y\right)$, where $y\in \{\mathrm{0,1}\}$ indicated whether an observation came from the real or synthetic dataset. Disclosure risk was deemed low if the classifier’s accuracy did not exceed a predefined threshold (e.g., the model performance was close to random guessing).

Identity disclosure was assessed in six (32%) studies. In this setting, an adversary aims to link a synthetic record ${x}^{*}$ to a real record $x$. In practice, all studies measured a distance $d\left(x,{x}^{*}\right)$ between original and synthetic records and counted re-identification when the nearest real record fell within a given threshold. If several records shared the same minimum distance, the match was treated as indeterminate. The calculation of this distance, however, varied: some studies used weighed distances and compared them to a dataset with one individual excluded (similar to leave-one-out cross-validation), while others calculated distances directly between the datasets.

Attribute disclosure was the second most frequently assessed aspect of privacy, examined in nine studies (47%). In this setting, an adversary is assumed to know a subset of variables ${X}_{A}$ for an individual and to use the synthetic dataset to infer the unknown sensitive attributes ${X}_{B}$. Disclosure is considered to occur if $P\left({X}_{B}|{X}_{A},{D}_{syn}\right)$ is sufficiently high compared to chance. In practice, threat models quantified disclosure risk either by measuring attribute matches between original and synthetic datasets using a predefined distance metric, or by training predictive models on synthetic and original data to assess the likelihood of observing particular attribute combinations or values.

## Discussion

To address RQ1, we identified 36 studies presenting methods able to generate synthetic longitudinal patient data, with the majority published in 2023. Of the 39 primary methods, 20 were mentioned in earlier medical SDG reviews and related methodological papers [17–25, 27], with the number in parenthesis indicating how many of the ten publications included each method: AC-GAN (5), ADS-GAN (4), CRBM (2), CTGAN (1), DSG (1), EHR-M-GAN (1), EVA (2), HALO (1), HealthGAN (7), LS-EHR (1), MTGAN (2), MultiNODEs (1), PromptEHR (3), SynTEG (6), Synthea (3), synthpop (2), TC-MultiGAN (1), TVAE (1), TWIN (1) and VAMBN (1). The difference in the number of methods identified in our review compared to earlier reviews cannot be fully explained by our inclusion of more recent publications as a significant portion of the methods from the overlapping years were either not recognized or were mentioned in only one of the earlier reviews. This likely results from variations in the databases and search strategies used, as well as the fact that some earlier reviews focused on specific methods like GANs or on different data types.

In addressing RQ2, our review differs from the earlier ones by providing a comprehensive and in-depth discussion and classification of the identified SDG methods from the perspective of longitudinal patient data. Specifically, we investigated how these methods address the temporal dimension of LPD and their ability to generate unbalanced LPD—topics that have not been properly addressed in previous reviews. Longitudinal patient data, in general, remain underexplored in the SDG literature, where terms like “time-series”, “trajectory”, or simply “EHRs” are often used interchangeably, highlighting the challenge of distinguishing between various time-dependent data types. Our findings reflect this difficulty, with approximately two-thirds of the methods focusing on generating standard longitudinal patient data and the rest on sequential or trajectory-based data (see Sect. 2.7). Given the overlap of these latter two types with time series, readers seeking methods for generating such data may find it useful to consult also the literature on synthetic time series generation.

Most identified methods were deep learning (DL) models. These models capture complex patterns from data without requiring strict distributional assumptions. However, DL models typically entail numerous training parameters and demand substantial sample sizes, limiting their use with small datasets. Dealing with multiple variable types is challenging and often necessitates variable encoding and normalization, reducing information and increasing dimensionality. Moreover, the effectiveness of DL models in generating longitudinal patient data relies heavily on their ability to discern patterns within the input data, yet they typically struggle with missingness and generating unbalanced observations. In the context of synthetic LPD generation, a step forward would involve developing and integrating components specifically designed to preserve temporal structures and generate unbalanced data, as seen in two novel approaches, cLSTM [27] and EHR-Safe [56], identified in this review.

Language models have made progress in addressing missingness and unbalance in LPD. However, it remains unclear whether missing values arise from the inherent unbalanced structure of LPD or if models are learning patterns of missingness and its distribution. Furthermore, the current implementations often require sample sizes in the tens of thousands and considerable computational resources, far exceeding those of traditional DL models. This makes them more suitable for large hospital databases than for study-specific datasets. Additionally, many implementations lack privacy-preserving mechanisms, which further limits their applicability. A more detailed discussion of how deep learning and language models are used in synthetic medical data generation can be found in [20].

In response to RQ3, we examined how the identified methods were evaluated in terms of resemblance, utility, and privacy preservation. While nearly all studies assessed resemblance, only every fifth evaluated all four domains: univariate, bivariate, multivariate, and temporal preservation. It is encouraging to see that the evaluation of temporal preservation, which we consider essential when generating synthetic LPD, has become more prevalent in recent studies (Fig. 4, panel 4). However, most studies still relied on subjective qualitative techniques. Temporal preservation could be more rigorously assessed using quantitative methods, such as comparing autocorrelations or transition matrices. Moreover, the relationship between categorical and numerical variables remains largely unexplored.

The evaluation of utility was even less frequent, with only three-fourths of studies addressing it, and there was a clear distinction between statistical inference and prediction tasks, with most studies focusing exclusively on the latter and none addressing both. This raises concerns about the reliability of statistical utility of the synthetic data generated by these methods. For example, although there is growing emphasis on developing predictive models using EHR data, much of medical research is aimed at advancing scientific knowledge by inferring (causal) mechanisms underlying the studied phenomena, something many predictive models are not designed to capture.

We note that resemblance and utility have overlap in their methodologies. For example, a t-test can be used to measure both distributional resemblance and to conduct inference. However, in this work, we distinguished between the two by defining resemblance as the comparison of the full distributions of the original and synthetic data (for practical reasons, often resorted to surrogates such as mean and variance comparisons). In contrast, utility was concerned only with comparing the values of a single model parameter (vector), without regard to the similarity of the full distributions in the data.

We observed no clear trends in the integration of privacy-preserving techniques within SDG, as only one in five methods implemented such measures. Furthermore, only half of the studies included a privacy evaluation, a pattern also noted by [23]. This limited focus on privacy may have reflected several factors: first, an implicit assumption that the use of randomized generation methods would by itself guarantee privacy; second, the practical challenges of implementing and evaluating privacy protections; and third, the absence of established guidelines for defining and measuring privacy thresholds.

The second issue, practical challenges in implementation and evaluation, was apparent in several ways. Implementing privacy-preserving techniques is difficult and requires careful application. Some implementations have been shown to fail consistently in upholding their theoretical privacy guarantees [5] and selecting an appropriate privacy budget remains a challenge [89]. These problems are amplified in longitudinal data, where each subject has multiple measurements, which may explain why such techniques were rarely applied.

In the context of LPD, privacy-preserving metrics must be used with particular care. While the reviewed studies did not elaborate on data formatting, we assume, based on the used metrics and attack models, that data were represented in wide format (see Fig. 1 panel c), where each patient occupies a single row and rows are treated as independent. This assumption is crucial as using the same metrics on data in long format (with multiple rows per subject) without accounting for repeated measurements can lead to misleading results. For example, calculating Euclidean between-row distances could inadvertently measure within-subject variation, and attack models might falsely treat repeated measurements as independent entities, likely inflating the number of false positives.

Another manifestation of practical challenges was that studies used similar approaches to assess SD quality and disclosure risk, making it unclear what was actually being evaluated. For instance, when multivariate resemblance was assessed using a model-based paradigm and the fitted model could not distinguish between original and synthetic data, this simultaneously suggested resemblance while also indicating no related disclosure risk. In this review, we classified each approach according to how it was framed by the study authors (i.e., whether they described it as an assessment of resemblance or privacy).

Given the absence of clear guidelines, we contend that evaluating SD for potential disclosure risks, particularly identity disclosure, is crucial because it leads to subsequent disclosures. Once an individual is identified, their membership in the dataset is automatically confirmed, and sensitive attributes associated with them are also revealed. Most studies assessed membership disclosure but not identity disclosure, possibly due to closeness of the two concepts or based on the assumption that if membership cannot be inferred, identity disclosure is unlikely to occur. However, this assumption rests on the membership disclosure being assessed correctly, which is not always the case. Recent work [90] has demonstrated that commonly used privacy-preserving methods and similarity-based privacy metrics can be misapplied, resulting in misleading disclosure risk estimates. Consequently, SD that was initially deemed private was later shown to be vulnerable, with membership, attribute and reconstruction attacks proving successful. Yet, these findings do not invalidate similarity-based metrics as a concept; rather, they highlight the need for context-aware evaluation and carefully defined similarity criteria, which is particularly critical for complex, temporally structured data such as LPD.

Importantly, the absence of identity disclosure does not preclude the possibility of membership disclosure. Membership disclosure can arise when patterns in the synthetic data allow an attacker to infer that certain types of individuals, or cases with specific characteristics, must have been included in the training data, even if no individual can be uniquely identified. Such risks are particularly concerning when the dataset relates to a sensitive topic, such as the presence of a specific disease. Therefore, we recommend systematically evaluating both identity and membership disclosure, while also encouraging the exploration of additional disclosure scenarios.

Of particular concern is that only one in ten studies comprehensively evaluated resemblance, at least one dimension of utility, and one aspect of privacy, casting doubt on the real-world applicability of the identified methods. This gap is likely due to the absence of a standardized evaluation framework, as highlighted by previous reviews [17–19, 21–23, 26]. While some frameworks are available [91–93], the third of which focuses entirely on privacy, these are largely based on cross-sectional data and do not adequately address other data types, such as LPD.

Our classification of resemblance, utility and privacy provides a foundation for developing a more comprehensive evaluation framework. Although some approaches are applicable to many different data types, our categorization facilitates the integration of data-specific (e.g., temporal structure) and task-specific utility metrics, accommodating both statistical and machine learning perspectives. However, the detailed specification of these metrics remains a topic for future research. Additionally, a crucial part of any evaluation is to use independent replications and average the metrics across them. If only a single synthetic dataset is used, it is possible that any observed outcome has occurred by chance alone. For reliable conclusions, the required number of replicates is likely in tens/hundreds. Surprisingly, only one in ten studies employed more than one replication.

Applications of longitudinal patient data, such as disease trajectory modeling, treatment effectiveness estimation, and personalized treatment planning, require extensive research, development, and innovation. While real-world LPD remain essential for these activities, synthetic LPD provide a powerful tool to accelerate RDI, allowing researchers to make progress while awaiting real data. Furthermore, synthetic LPD could be utilized in medical education and training, providing realistic patient scenarios for students and practitioners to explore without compromising patient confidentiality. Our results demonstrate that multiple methods for generating synthetic LPD exist. Despite the availability of these methods, the widespread use and sharing of synthetic datasets remain limited. Factors such as the lack of standardized evaluation frameworks and ongoing concerns about the sufficiency of privacy protections likely contribute to this limitation.

None of the previous reviews [17–27] systematically assessed the risk of bias or reporting quality, although [22] recognized the potential for biases. Thus, our review appears to be the first in this regard. Despite our structured framework and validation by all authors, these assessments remain somewhat subjective, a recognized characteristic even within established frameworks [94]. Approximately every third publication lacked a transparent description of comparison procedures (risk of performance bias), and selective reporting (risk of reporting bias) was present almost in half of the studies. Furthermore, like [18], we identified inadequacies in the training process descriptions and source code availability. Our findings are not meant to criticize any single study, and any shortcomings are most likely oversights. The review’s findings should be regarded as overarching recommendations for improving the transparency of SDG research.

To reduce the risk of bias and improve reporting quality in future SDG research, we encourage authors to provide clear documentation of data preprocessing as well as training and evaluation procedures, including but not limited to how missing data or unbalancedness were handled, what formatting was required (i.e., long or wide), methods of variable discretization, reporting whether generated values fall within the range of the original data (an aspect that was generally not described in the reviewed studies), and the application of benchmarking methods. While all reviewed methods were applied to longitudinal data, this level of transparency is especially important given that several reference methods were not originally developed for such contexts. It remained often unclear how these methods were adapted to account for temporal structure, which raises concerns about the fairness and validity of comparative evaluations. Authors should ensure that evaluation conditions are standardized across methods or, when differences are unavoidable, explicitly acknowledge and justify them.

To support reproducibility and implementation, we strongly encourage the sharing of source code, evaluation scripts, and synthetic datasets. All evaluation procedures described in the methods section should be reported in the results or supplementary materials, regardless of outcome. Statistical reporting should follow consistent notation and include sufficient detail, such as sample sizes, model parameters, p-values, confidence intervals, and privacy budgets where applicable. Additionally, system-level information, such as memory usage, graphical processing unit requirements, and runtime, was often missing in the reviewed studies but is critical for users seeking to apply these methods within their resource constraints. Lastly, authors should disclose funding sources and any potential conflicts of interest to promote transparency and trust in the field.

### Limitations

Although our review, to our best knowledge, is the first systematic literature review on SDG that adheres thoroughly to the PRISMA guidelines (checklist in Supplementary material A.9), including the preparation and submission of a review protocol (CRD42021259232) to the international systematic review registry PROSPERO [95], it is important to acknowledge its inherent constraints.

First, due to inconsistent definitions of synthetic and longitudinal patient data as well as the diverse evolution of SDG across various fields, formulating a definitive search algorithm was challenging, leading to several false positives during the screening phase (see Fig. 2). Despite our efforts to encompass synonyms, omitting relevant publications remains possible. Nevertheless, given the large number of screened publications and exhaustive citation searching, coupled with our accurate identification of intersecting publications observed in the prior reviews, we are confident in the comprehensiveness of the literature included.

Second, longitudinal data analysis has mainly been used in medical statistics, while SDG methods derive predominantly from computer science and associated applications. This discrepancy poses challenges in assessing the applicability of SDG methods for LPD generation. For instance, the notion of unbalanced data, though well-established in statistics, has received limited attention in computer science, resulting in its underrepresentation in SDG research. This likely explains why the respective information was not available in the included studies (Table 3).

Third, it is crucial to note that scientific advancements continue and this review is confined to data accessible until May 2024. Since most of the methods identified in this review were published from 2022 onward, it is likely that new methods have already been introduced. Our findings suggest that these newer methods are likely to address issues related to the unbalanced nature and missingness of LPD, and we anticipate that privacy-preserving mechanisms are being integrated into these approaches. Nevertheless, we believe that our work provides a solid foundation by presenting 39 methods. This number increases to 66 when including the 27 reference methods, offering researchers and data controllers a comprehensive selection of approaches to meet their specific needs, while also highlighting current shortcomings and suggesting practical areas for improvement in future method development.

Finally, methodological reviews typically perform empirical evaluations across methods to enable direct comparisons. However, in this review such an approach was not feasible due to several practical challenges. The reviewed methods differed substantially in their requirements for data preprocessing, such as handling of missing data, unbalanced longitudinal structures, and mixed variable types (Table 3). Even when grouped by similar characteristics, method-specific adaptations would have been needed, resulting in inconsistencies in the training data that limit the fair comparability of results. In addition, many methods lacked publicly available source code and re-implementing methods independently was beyond the scope of this review. As a result, our study provides a structured overview but only a limited foundation for selecting one method over another in applied settings. We therefore encourage future work to complement this review with experimental comparisons and use-case–oriented recommendations.

### Conclusion

Our review identified 39 methods for generating synthetic longitudinal patient data (RQ1), addressing various challenges associated with longitudinal patient data (RQ2). Only EHR-Safe [56], DSG [47], HALO [55] and cLSTM [27] addressed all challenges simultaneously, but none incorporated privacy-preserving mechanisms, and all relied on resource-intensive deep learning or language models. Furthermore, the quality of evaluating synthetic data varied significantly across studies (RQ3), leaving the methods’ real-world applicability uncertain, especially across diverse datasets. These findings highlight a critical gap and underscore the need for developing more robust, efficient, and privacy-preserving synthetic data generation methods for longitudinal patient data.

Due to the many different forms of LPD, no single method is likely to cover all cases, and more focused approaches are advised. For example, for standard longitudinal patient data one could consider a hybrid approach where static covariates are generated using an assumption-free generative model and unbalanced time-varying responses are modelled conditionally on the covariates using hierarchical models that acknowledge the missingness and time-dependence in the measurements. Similarly, methods producing partially synthetic LPD, such as [96], could possibly be combined with additional generative models to achieve novel full SDG methods.

The observed heterogeneity in the evaluation approaches across studies presents a significant challenge in conducting comparisons between methods and their applicability in practice. While creating standardized evaluation criteria would enhance method assessment, recognizing the importance of tailored approaches for various applications is crucial. Establishing a standardized evaluation framework offers a chance for interdisciplinary collaboration among medicine, statistics, and computer science. Our categorization of different evaluation approaches for assessing resemblance, utility and privacy provides a robust basis for subsequent research.

Lastly, further research is needed to address privacy concerns surrounding SD, along with clear guidance from data protection authorities, such as official standards for defining privacy thresholds for data publication, possibly in the context of a suitable privacy framework such as [93]. Currently, there is a gap between the development of SDG methods and the availability of synthetic patient data and platforms to facilitate their use. Bridging this gap will require collaboration among method developers, medical practitioners and policymakers as the directives will require empirical support and new methods should be developed with practical feasibility in mind. Overcoming these challenges will help unlock the full potential of synthetic data in transforming healthcare.

## Supplementary Information

Below is the link to the electronic supplementary material.

Supplementary file1 (PDF 2948 KB)

## Data Availability

The data used to derive the results and conclusions of this systematic review are available upon request.
