# Supplementary material for: Methods for Generating and Evaluating Synthetic Longitudinal Patient Data: A Systematic Review
Source: J Healthc Inform Res. 2025 Nov 17;10(1):1–39. doi: 10.1007/s41666-025-00223-7 (PMC12872944; doi:10.1007/s41666-025-00223-7)
Supplement: Supplementary file 1 — Supplementary file1 (PDF 2948 KB) [file 41666_2025_223_MOESM1_ESM.pdf]

Supplementary material for "Methods for generating and evaluating synthetic longitudinal patient data: a systematic review"

A.1 Search algorithms

A.1.1 Web of Science (Core Collection)

Search date 2021-06-11, 3795 hits

```
#1 TS = ((synthetic OR artificial)
NEAR/3 (*data* OR record*))
AND TS = ((generat* OR produc* OR simula*))
AND TS = ((longitudinal OR correl* OR panel
           OR repeat* OR follow-up
           OR multivariate OR lifespan*
           OR traject* OR health*
           OR medical OR patient))
NOT TS = (aperture OR insemination OR seism*)
AND LA = (English)
AND DT = (Article OR Abstract of Published Item
          OR Book OR Book Chapter OR Data Paper
          OR Early Access OR Proceedings Paper
          OR Review OR Software Review)
```

Search date 2022-11-22, 1734 hits

```
#2 TS = ((synthetic OR artificial)
NEAR/3 (*data* OR record*))
AND TS = ((generat* OR produc* OR simula*))
AND TS = ((longitudinal OR correl* OR panel
           OR repeat* OR follow-up
           OR multivariate OR lifespan*
           OR traject* OR health*
           OR medical OR patient))
NOT TS = (aperture OR insemination OR seism*)
AND LA = (English)
AND DT = (Article OR Abstract of Published Item
          OR Book OR Book Chapter OR Data Paper
          OR Early Access OR Proceedings Paper
          OR Review OR Software Review)
NOT #1
```

Search date 2024-05-21, 1278 hits
Like 2022-11-22 but limited to results published after 2022-11-22

A.1.2 Embase (1947 onwards)

Search date 2021-06-11, 504 hits

```
#1 (((synthetic OR artificial)
NEAR/3 (data OR record*)):ti,ab,kw)
AND (generat* OR produc* OR simula*):ti,ab,kw
AND (longitudinal OR correl* OR panel
OR repeat* OR ’follow?up’
OR multivariate OR lifespan*
OR traject* OR health* OR medical
OR patient):ti,ab,kw
AND ([article]/lim OR [article in press]/lim
OR [conference paper]/lim
OR [conference review]/lim
OR [data papers]/lim OR [letter]/lim
OR [note]/lim OR [review]/lim
OR [short survey]/lim)
AND [english]/lim
AND [embase]/lim
```

Search date 2022-11-22, 326 hits

```
(((synthetic OR artificial)
NEAR/3 (data* OR record* OR microdata*)):ti,ab,kw)
AND (generat* OR produc* OR simula*):ti,ab,kw
AND (longitudinal OR correl* OR panel OR repeat*
                           OR 'follow?up' OR multivariate OR lifespan*
                           OR traject* OR health* OR medical OR patient):ti,ab,kw
NOT (aperture OR insemination OR seism*):ti,ab,kw
AND ([article]/lim OR [article in press]/lim
                           OR [conference paper]/lim OR [conference review]/lim
                           OR [data papers]/lim OR [letter]/lim OR [note]/lim
                           OR [review]/lim OR [short survey]/lim)
AND [english]/lim NOT #1
```

Search date 2024-05-21, 314 hits
Like 2022-11-22 but limited to results published after 2022-11-22

A.1.3 MEDLINE (Ovid interface, 1946 onwards)
Search date 2021-06-12, 574 hits

```
#1 (((synthetic or artificial)
    adj3 (data or record*))
    and (generat* or produc* or simula*)
    and (longitudinal or correl* or panel
        or repeat* or 'follow up'
        or multivariate or lifespan*
        or traject* or health* or medical
        or patient)).ti,ab,kf.
 #2          limit #1 to ((english language or english)
    and (classical article or clinical conference
        or comparative study or congress
        or english abstract or evaluation study
        or festschrift or government publication
        or historical article
        or introductory journal article
        or journal article
        or letter or preprint or "review"
        or "systematic review" or technical report
        or validation study))
```

Search date 2022-11-22, 402 hits (contains duplicates with the previous search because the time range could not be specified more precisely)

```
#3 (((synthetic or artificial)
    adj3 (data* or record* or microdata*))
    and (generat* or produc* or simula*)
    and (longitudinal or correl* or panel
        or repeat* or 'follow up'
        or multivariate or lifespan*
        or traject* or health* or medical
        or patient)
    not (aperture OR insemination OR seism*)).ti,ab,kf
#4 limit #3 to ((english language or english)
    and (classical article or clinical conference
    or comparative study or congress
    or english abstract or evaluation study
    or festschrift or government publication
    or historical article
    or introductory journal article
    or journal article or letter or preprint
    or "review" or "systematic review"
```

```
    or technical report or validation study))
#5 limit #3 not #2
```

Search date 2024-05-21, 355 hits
Like 2022-11-22 search.

A.1.4 Google Scholar (Publis or Perish software, 1000 first hits)

Search date 2021-06-18, 980 hits

```
("synthetic data" OR "artificial data")
AND (generat* OR priduc* OR simula*)
AND (longitudinal OR correl* OR panel
    OR repeat* OR "follow up" OR "follow-up"
    OR "multivariate OR lifespan* OR traject*
    OR health* OR medical OR patient)
```

The search was not repeated because Google Scholar flagged the software as a bot, leading to a temporary ban.

A.1.5 arXiv
Open-source metadata were downloaded from Kaggle [18] and R software (version 4.2.2) [71] was used to extract the relevant articles. The source code is presented below.

Search date 2022-11-22, 628 hits

```
# libraries
library(jsonlite)
library(data.table)
library(synthesisr)

# importing ArXiv results
arxiv <- stream_in(file(paste0(getwd(), "/articles/source_searches/arxiv-metadata-oai-snapshot.json")))
arxiv <- as.data.table(arxiv)

# regex developed according to database search queries
# synthetic data
search_data <-"\\b(synthetic|artificial)(?:\\W+\\w+){0,3}?\\W?(\\S*data|record\\S*)\\b"

# inclusion criteria
search_gener <- "(generat|produc|simula)"
search_type <- "(longitudinal|correl|panel|repeat|follow-up|multivariate|lifespan|traject|health|medical|patient)"

# exclusion criteria
search_excl <- "(aperture|insemination|seism)"

# grepping abstracts according to criteria
```

```
arxiv_results_1 <- arxiv[grepl(search_data, abstract, ignore.case = T, perl = T)]
arxiv_results_2 <- arxiv_results_1[grepl(search_gener, abstract, ignore.case = T, perl = T)]
arxiv_results_3 <- arxiv_results_2[grepl(search_type, abstract, ignore.case = T, perl = T)]
arxiv_results_4 <- arxiv_results_3[!grepl(search_excl, abstract, ignore.case = T, perl = T)]

# modifying data for export
arxiv_results_4[, source_type := ifelse(is.na(`journal-ref`), "UNPB", "JOUR")]
arxiv_results_4[is.na(`journal-ref`), `journal-ref` := paste0("arXiv preprint arXiv:", id)]
arxiv_results_4[, year := year(update_date)]

setnames(arxiv_results_4, "journal-ref" , "journal")
setnames(arxiv_results_4, "update_date" , "date_generated")
setnames(arxiv_results_4, "authors" , "author")

arxiv_results_4[, c("id", "submitter", "comments", "report-no",
                    "categories", "license", "versions", "authors_parsed") := NULL]

setcolorder(arxiv_results_4, c("date_generated", "source_type", "author",
                               "year", "title", "journal", "doi"))

arxiv_results_4[, author := gsub(",", " and", author)]
arxiv_results_4[, author := gsub("\n", "", author)]
arxiv_results_4[, author := gsub("\\\\", "", author)]
arxiv_results_4[, author := gsub("\\"", "", author)]
arxiv_results_4[, author := gsub("[(]\\d[)](\\W?and)?", "and", author)]

# exporting as ris file
write_refs(as.data.frame(arxiv_results_4), format = "ris", file = paste0(getwd(),
"/articles/source_searches/arxiv_results.ris"))
```

Search date 2024-05-21, 417 hits
Similar to 2022-11-22, limited to results published after 2022-11-22.

A.2 Selection process

A.2.1 Abstract screening chart

Each included search result was screened by KP and JV independently using Rayyan [67] and the flowchart presented below. The process started at the top of the chart (Start) where titles and abstracts were first assessed for evidence of data generation. If this could not be determined with confidence, the record was classified as **Maybe** and proceeded to full-text screening for confirmation.

If a record was judged to involve data generation, the second step assessed whether the generated data were applicable to longitudinal patient data. Records using the wrong data type were given a corresponding exclusion label (the list of possible labels expanded as the screening progressed).

In the third step, we evaluated whether the generated longitudinal data met our definition of synthetic data. For this review, synthetic data were defined as data generated via a randomized algorithm applied to an existing dataset (original or input data), with the goals of closely mimicking the original data distribution and enabling the creation of an unlimited number of synthetic samples. Records were excluded if the data were generated instead by:

- 3a) Data simulation – generation from theoretical models or algorithms based only on standard probability distributions and taking no real data as input.
- 3b) Resampling / permutation methods – such as bootstrap or shuffling of existing values.
- 3c) Deterministic algorithms, e.g., rule-based methods (“if this condition, then that value”) or procedures lacking any stochastic component.

In cases of continued uncertainty, the document proceeded to full-text screening for clarification. At each stage, the process advanced according to the arrows in the chart, with the final decisions (**Include, Maybe, Exclude**) indicated in bold.

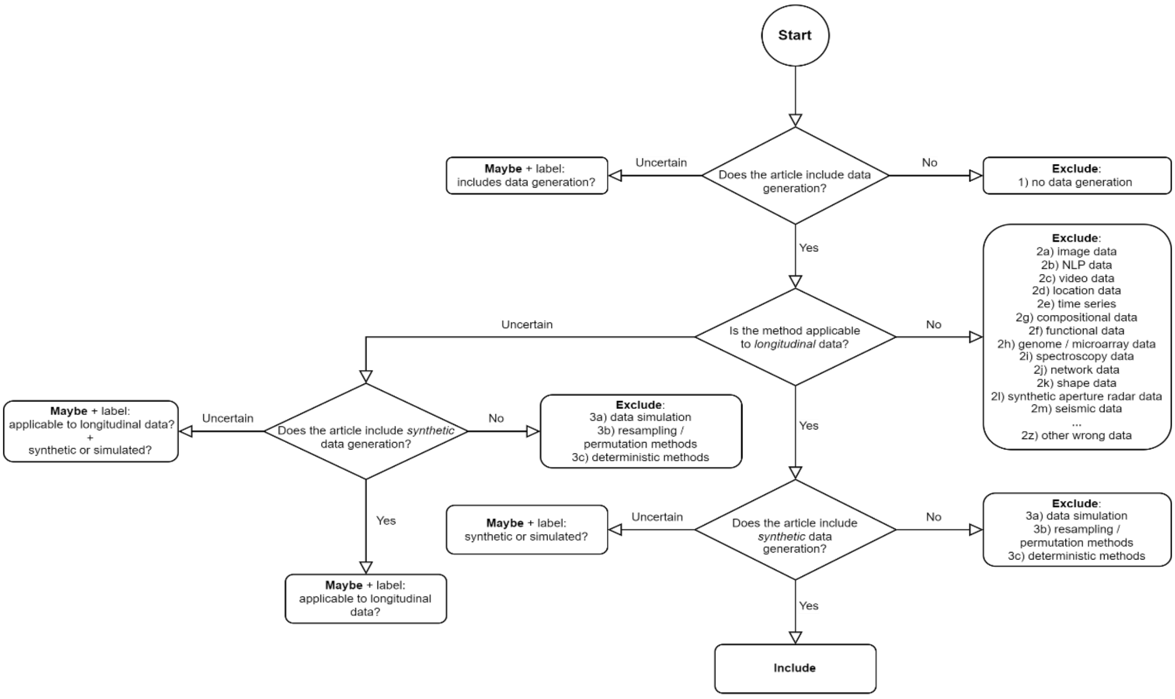

A.2.2 Full-text screening chart

Full texts of each publication that was included after screening the titles and abstracts were screened by KP and JV independently using Rayyan [67] and the flowchart presented below. The process started at the top of the chart (Start) by confirming whether the record contained synthetic data generation. This was often the reason why records had been classified as **Maybe** during abstract screening, since this aspect was not always evident from the abstract.

At this stage, we excluded records that did not meet our synthetic data criteria (1a; see Section 2.1 in the main text, also marked with an asterisk in the chart below). We assigned separate labels to methods based on standard probability distributions (1b) and those using partially synthetic data (1c).

Next, we assessed applicability to longitudinal patient data, based on the criteria (see Sections 1 and 2.1 in the main text, also marked with double asterisks in the chart below). At this step, different data types were not separated; records were excluded if applicability could not be confirmed.

In the third step, we evaluated whether description of the data-generating method was available, as our review focused on identifying reproducible methods. Records without documentation of the data generation process were excluded (3a). Finally, we confirmed that the method was sufficiently described to be reproducible, either by re-implementation from pseudocode or equivalent documentation, or by direct use of available source code.

The process followed the arrows in the chart, with final decisions (**Include, Maybe, Exclude**) indicated in bold.

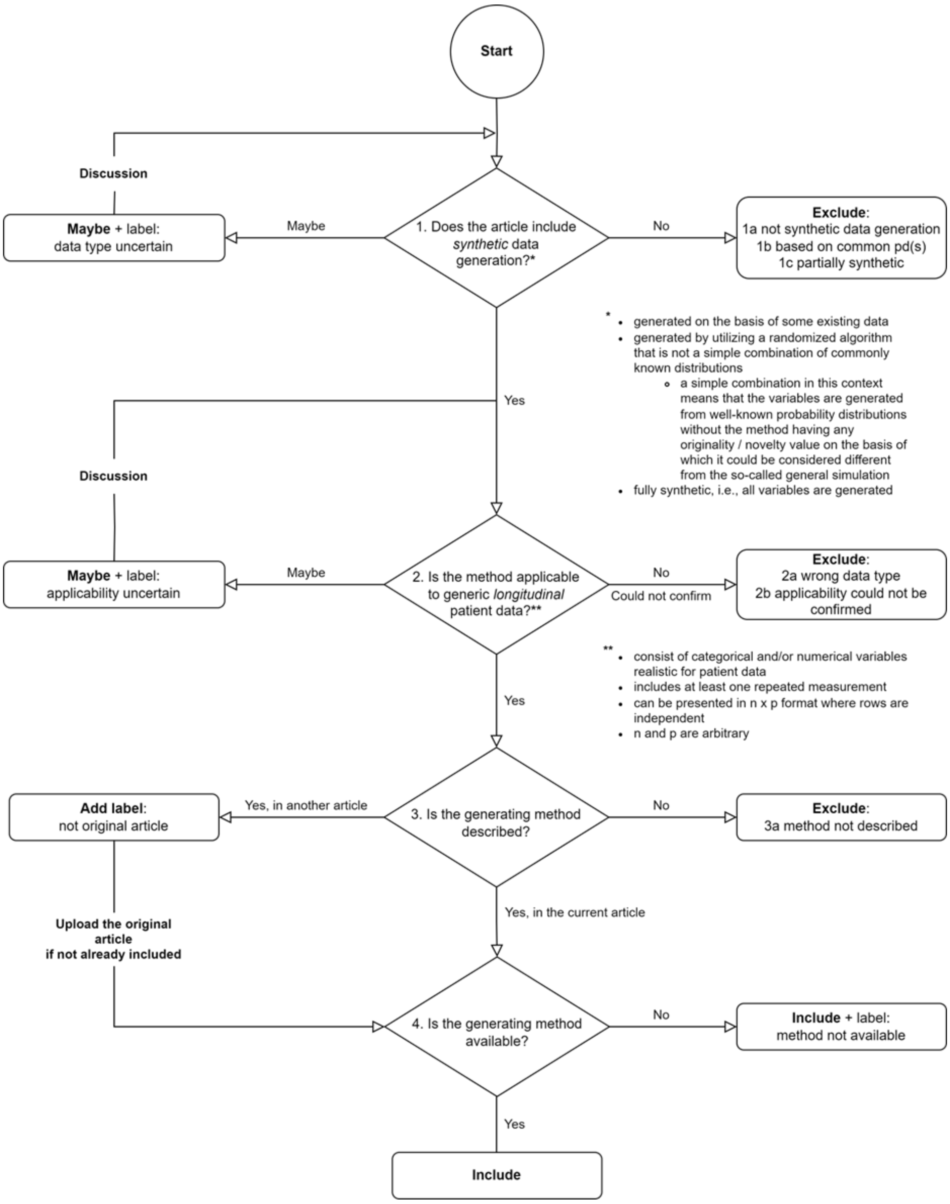

A.3 Data collection process

Data were collected and managed by the corresponding author using a structured form designed in REDCap electronic data capturing tools hosted at University of Turku [35, 36]. The forms are presented below.

A.3.1 Literature information

The results of Literature information collection are presented in Section 3.2 of the main text.

Page 1

Literature information

Please complete the survey below.

Type of publication	◯ Journal article<br>◯ Poster<br>◯ Conference paper<br>◯ Book chapter<br>◯ Dissertation or thesis<br>◯ Research report<br>◯ Review article<br>◯ Other
Name the other publication type	__________________________
Authors	________________________________
Year	__________________________
Title	__________________________
Publication platform (journal, conference, book...)	__________________________<br>(Give the name of the journal/conference etc.)
Volume	__________________________
Issue	__________________________
Page numbers	__________________________
Is the publication peer-reviewed?	◯ Yes<br>◯ No
What was the purpose of the study?	________________________________<br>(In this context, study refers to the article)

11.08.2023 11:27 projectredcap.org REDCap®

A.3.2 Method characteristics

The results of the method characteristics data collection are presented in Section 3.4 of the main text. Certain details, such as software license, system requirements, and value ranges, were inconsistently reported in the identified literature and therefore are not systematically presented in the main results. These issues are discussed in Section 4, paragraphs 17–19.

Page 1

Method characteristics

Please complete the survey below.

**Basic information**	
Type of the method	☐ Generative adversarial network<br>☐ Recurrent neural network<br>☐ Auto-encoder (variational or other)<br>☐ Bayesian network<br>☐ Hidden Markov model<br>☐ Density estimation<br>☐ Imputation method<br>☐ Dimensionality reduction<br>☐ Data partitioning<br>☐ Decision tree (classification, regression)<br>☐ Posterior predictive sampling<br>☐ Clustering<br>☐ Other deep learning method<br>☐ Other
Type of the method (other/other deep learning)	__________________________
Is expert knowledge required/used in the method?	◯ Yes<br>◯ No
How is expert knowledge needed?	________________________________
Describe the method as concisely as possible	________________________________
Programming language	☐ R<br>☐ Python<br>☐ C++<br>☐ Java<br>☐ Scala<br>☐ Julia<br>☐ Fortran(77/90/95/...)<br>☐ Matlab/Octave<br>☐ SAS<br>☐ Other<br>☐ Not specified
Programming language (other)	__________________________
Is the pseudocode of the method presented?	◯ Yes<br>◯ No
Is the source code/software provided?	◯ Yes<br>◯ No<br>◯ Upon request

11.08.2023 11:27 projectredcap.org REDCap®

Method's source code location	__________________ (e.g. URL)
Is the software	◯ Library<br>◯ Standalone software<br>◯ Other<br>◯ Not specified
Define the other software type	__________________
Is the software used to apply the method free?	◯ Yes<br>◯ No<br>◯ Not specified
Software licence	__________________
Was the method	☐ Originally desinged for longitudinal data<br>☐ Altered/modified for longitudinal data<br>☐ Implemented to longitudinal data without any modifications
How the method model/approaches longitudinal data	__________________

Used system and complexity (requirements)

Does the article mention anything about the used system or its requirements?	◯ Yes<br>◯ No
Operating system	__________________
Other system requirements or used system information	__________________
Method's running time in terms of the input size (Big O notation) if reported	__________________

Input (original) and output (synthetic) data properties

Is the method capable of handling unbalanced longitudinal data?	◯ Yes (number of time points / timing / spacing for intervals is different for different subjects)<br>◯ Yes (some variables are collected less often than others, but still for everyone at the same time point)<br>◯ No<br>◯ Not specified

Is the method capable of generating unbalanced longitudinal data?	◯ Yes (number of time points / timing / spacing for intervals is different for different subjects)<br>◯ Yes (some variables are collected less often than others, but still for everyone at the same time point)<br>◯ No<br>◯ Not specified
The method is capable of	☐ Handling categorical original data<br>☐ Handling numerical original data<br>☐ Generating categorical synthetic data<br>☐ Generating numerical synthetic data<br>(numerical = continuous / interval, categorical = binary / multiclass)
The numerical data values generated	☐ Will not necessarily fall within the corresponding range in the original data set<br>☐ Will fall within the corresponding range in the original data set<br>☐ Will be replicates of values in the original data set<br>☐ Not specified
Is the method capable of handling missing values in the original data?	◯ Yes<br>◯ No<br>◯ Not specified
Is the method capable of producing missing values for synthetic data?	◯ Yes<br>◯ No<br>◯ Not specified

A.3.3 Method evaluation

The results of the Method performance evaluation data collection are presented in Section 3.5 of the main text, with the datasets provided in Supplementary Section A.7. Certain information, such as missing data patterns or the handling of repeated measurements as response/explanatory variables, was rarely reported in the included studies. Reported advantages and disadvantages of the methods are summarized to support a general discussion of the different method categories in Section 4 of the main text.

Method performance evaluation

Please complete the survey below.

**Data used to generate synthetic data, i.e., original or input data**	
Synthetic data was generated based on	☐ Real-world data<br>☐ Simulated data<br>☐ Synthetic data<br>☐ Other<br>(i.e., what type/form was the original data?)
Give the name of the data set(s)	____________________<br>(Separate the names with a comma)
What kind of data was used? Separate different data sets with a comma.	____________________<br>(e.g., patient data, other data related to people, non-human data)
Is the used data set(s) available?	☐ Publicly<br>☐ Upon request<br>☐ No
Give the source(s) of the available data set(s)	____________________<br>(If multiple, in same order as given the data sets above)
The number of independent observations (subjects) in the input data set(s). If multiple data sets, separate with a comma.	____________________<br>(If not reported or recoverable, write 'Not specified')
The number of variables in the input data set, including variables with repeated measurements. If multiple data sets, separate with a comma.	____________________<br>(If not reported or recoverable, write 'Not specified')
Number of variables with repeated measurements (subset of the total number of variables).	____________________
Number of repeated measurements. For unbalanced data or varying ranges, give the range [min, max]. If multiple data sets are used, separate them with a comma.	____________________
Number of categorical variables. If multiple data sets are used, separate them with a comma.	____________________<br>(Options: 0,1,,2,..., not specified)
Number of numerical variables. If multiple data sets are used, separate them with a comma.	____________________<br>(Options: 0,1,,2,..., not specified)

Is the pattern of missingness similar to the original data?	◯ Yes<br>◯ No<br>◯ Not specified
Does the method have other limitations or requirements for the original data that have not already been mentioned?	◯ Yes<br>◯ No<br>(e.g. input data have to be scaled)
Describe the requirements / limitations regarding to original data	__________________________________

Evaluation setup of the generated synthetic data

Was the variable(s) in the synthetic data with repeated measurement treated as	◯ Response<br>◯ Explanatory<br>◯ Both<br>◯ Not specified<br>(Option "Not specified" should be used only if the article is otherwise relevant and the nature of the variable cannot be determined even through discussion.)
The evaluation of the generated synthetic data was based on	☐ Qualitative assessment<br>☐ Quantitative assessment<br>☐ Other<br>(Select all suitable options)
Describe the other approach used to evaluate the synthetic data and/or the method	__________________________________
The evaluation of the generated synthetic data was based on	☐ A single repetition (i.e., the assessment is based on a single generated synthetic data set)<br>☐ A small amount of repetitions (i.e., multiple data sets)(< 50)<br>☐ A large amount of repetitions (>= 50)<br>(Select all suitable options)
Was any of the following used to describe or evaluate the generated synthetic data and/or the method	☐ Descriptive statistics<br>☐ Statistical inference<br>☐ Prediction/classification (synthetic vs. real)<br>☐ Prediction/classification (some other variable)<br>☐ Privacy<br>☐ Externally assessed realism<br>(Select all suitable options)
Was the generated synthetic data evaluated	☐ Against (resamples) original data<br>☐ Against other simulated data<br>☐ Against other real-world data (public / private)<br>☐ Against another synthetic data set(s) generated by the same method (e.g., using different parameters)<br>☐ Against another synthetic data set(s) generated by a different method or methods<br>☐ No comparisons to other data or methods were made (i.e., a single data set was generated)<br>☐ Other<br>(Select all suitable options)

Name the other methods used in the comparison	______________________________
Describe the other approach to used to evaluate the generated synthetic data (in terms of data)	______________________________
Describe the simulation approach.	______________________________
Was the training process(es) described and/or available in the source code?	◯ Yes<br>◯ Partially<br>◯ No
What was lacking from the training process description if it was only partially described?	______________________________

Qualitative methods used to characterize and/or evaluate the generated synthetic data set(s)

Specify all qualitative methods (e.g., figures) used to describe and/or evaluate the generated synthetic data set(s)	______________________________

Descriptive methods used to characterize and/or evaluate the generated synthetic data set(s)

Specify all descriptive statistics (e.g., measures, estimates) used to describe and/or evaluate the generated synthetic data set(s)	______________________________

Inferential statistics used to evaluate the generated synthetic data set(s)

Specify all inferential statistics (e.g., tests, models) used to evaluate the generated synthetic data set(s)	______________________________

Predictive and classification approaches used to evaluate the generated synthetic data set(s)

Specify all the predictive and classification approaches (e.g., models, accuracy measures) used to evaluate the generated synthetic data set(s) in terms of synthetic data performance	______________________________

Privacy of the method and the generated synthetic data set(s)

Was differential privacy used to enhance/secure the privacy of the generated synthetic data?	◯ Yes<br>◯ No<br>(e.g., as a part of the method / applied post-hoc)
What was the epsilon used? If multiple epsilons were used, separate them with a comma	________________________
Specify delta if applicable. If value not specified, write not specified.	________________________

Was any of the following used to test the privacy of the synthetic data?	☐ Membership attack / identity disclosure<br>☐ Attribute disclosure<br>☐ Inferential disclosure<br>☐ Other<br>☐ No other approaches were used
Specify how the privacy of the method and/or the generated synthetic data set(s) was addressed: specify the approach (e.g., distinguishing records with a model) and parameters used (other than DP asked previously) if reported or write a summary of the authors' discussion on the subject if no specific approach was used.	__________________________________

Externally assessed realism

How was the realism assessed externally?	__________________________________

Other limitations or requirements for the generated synthetic data

Does the method have other limitations or requirements for synthetic data that have not already been mentioned?	◯ Yes<br>◯ No
Describe the requirements / limitations regarding to synthetic data	__________________________________

Advantages and disadvantages of the method

Did the authors discuss the advantages / disadvantages of the method?	◯ Yes<br>◯ No
Write down the advantages of the method according to the authors	__________________________________
Write down the disadvantages of the method according to the authors	__________________________________
Write down the advantages of the method according to you	__________________________________
Write down the disadvantages of the method according to you	__________________________________

A.3.4 Assessment of bias and reporting quality

The results of the assessment of bias and reporting quality are presented in Section 3.3 of the main text. The framework used to address the assessment questions is provided in Supplementary Section A.4.1, and the detailed results are available in Supplementary Section A.4.2.

Assessment of bias and reporting quality

Please complete the survey below.

For more information, see "Risk of bias in individual studies" in the review protocol.

Selection bias

Does the study show evidence of selection bias?<br><br>Assumption: The data used and the choice of model(s) should always be justified.<br><br>Examples:<br><br>Using a data set that is known in advance to perform poorly with another method that is used as a reference for the developed method Post hoc alteration of data or model inclusion based on arbitrary or subjective reasons Using different training, validation, or test sets when evaluating the method performance	◯ Yes<br>◯ No<br>◯ Possibly<br>(The option "Possibly" can be used in a situation where there is no clear evidence of bias, but there is something to point out about the subject.)
Describe the (possible) selection bias present	______________________________________

Performance bias

Does the study show evidence of performance bias?<br><br>Assumption: Method comparison procedures should be fair and carefully described.<br><br>Examples:<br><br>No fine-tuning is performed on the reference methods while the method in question is fine-tuned.	◯ Yes<br>◯ No<br>◯ Possibly<br>(The option "Possibly" can be used in a situation where there is no clear evidence of bias, but there is something to point out about the subject.)
Describe the (possible) performance bias present	______________________________________
In how many comparisons out of all reported comparisons did the method perform worse than another comparison method.	______________________________<br>(Give a fraction worse/total or write 0/1 if the method performed best/worst in every comparison)
List the situations in which the method performed worse than the other methods or write all and give the amount of comparisons reported, if the method performed worst in every comparison.	______________________________________

Reporting bias

Does the study show evidence of reporting bias?

Assumption: All metrics used in the study to evaluate the performance of the method should be described in the study and the results for these should be available to the reader.

Examples:

The performance of the method has been found to be measured in some way, but the results are only partially or not at all presented.

◯ Yes
◯ No
◯ Possibly
(The option "Possibly" can be used in a situation where there is no clear evidence of bias, but there is something to point out about the subject.)

Describe the (possible) reporting bias present

____

Inconsistency, imprecision and indirectness of reporting

Did the study show evidence of

☐ Inconsistency of reporting
☐ Imprecision of reporting
☐ Indirectness of reporting
☐ None of the above

Describe the type of inconsistency present

____

Describe the type of imprecision present

____

Describe the type of indirectness present

____

Competing interests

Were competing interests reported?

◯ Yes
◯ No
◯ Not available

A.4 Risk of bias and reporting quality assessment

A.4.1 Risk of bias assessment framework

The table below outlines the framework used to assess the risk of different biases that could influence the evaluation of the methods' performance in the included publications. It presents the fundamental principles (Rationale) that guided the assessment as well as the challenges involved in recognizing each type of bias (Assessment plausibility), along with illustrative examples of each type of bias.

Bias	Rationale	Assessment plausibility	Examples
Selection bias	Assessing the method's performance requires fairness in data representation, use of suitable metrics, and equal potential across methods to perform specific tasks. This necessitates clear justifications for input data, metrics, and reference method selection.	Detecting selection bias is difficult because any assessment approaches taken prior to the final publication may not be fully disclosed, making it difficult to assess favoritism towards the primary method. The reviewers may also be unaware of instances where a particular dataset did not work well with a particular method.	- Adjusting data or models based on arbitrary factors.<br>- Using different datasets to evaluate different methods<br>- Selectively using data or methods to favor the primary method.
Performance bias	To ensure a fair performance evaluation across methods, it is essential that a transparent and detailed description of the comparison and training procedures has been provided.	Detecting performance bias is challenging when the model selection and training details are incomplete or not reported. It becomes possible when the authors provide these details and mention using reference methods without task optimization.	- Not giving the reference methods a fair opportunity to perform well, e.g., through intentionally inadequate model training compared to the primary method.
Reporting bias	To ensure research transparency, it is important that all research evaluation metrics are comprehensively documented, and the results are shared.	Detecting the bias should be straightforward when a publication or its supplementary material lacks or incompletely presents results for the evaluation approaches mentioned in the study.	- Results are either incomplete or missing

A.4.2 Risk of bias in individual studies (detailed explanations)

The following table contains detailed explanations of the identified risk of bias within each study. The risk of bias was assessed using the criteria presented in Appendix A.4.1.

Authors	Performance bias	Explanation	Reporting bias	Explanation
Bhanot et al. [10]	Possibly	The method was compared to other methods, but the training processes were not described.		
Li et al. [54]	Possibly	The method was compared to other methods, but the training processes were not described.	Yes	Certain outcomes were exclusively or incompletely reported across methods and/or datasets. For instance, not all outcomes of t-tests were fully given, and patient trajectories were displayed only for the primary method and using only the MIMIC-III data.
Yu, He & Raghunathan [102]	Possibly	The method was compared to other methods, but the training processes were not described.	Yes	Certain findings, such as those shown in Table 2, pertained only to the primary method. Furthermore, the outcomes pertaining to IVEWare were excluded from the tabulated results of Tables 3 and 4. These specific outcomes were also omitted from the supplemental materials.
Zhang et al. [105]			Yes	The authors asserted in their work (page 602, top of the second column) that statistical insignificance of FPR and TPR was observed. However, we could not find information about the specific statistical test they used in this context.
Zhang, Yan & Malin [106]	Possibly	The method was compared to other methods, but the training processes were not described.	Yes	The primary method "Baseline + CFR + RS" was omitted from Figure 5 illustrating the drift in time.
Biswal et al. [11]	Possibly	The method was compared to other methods, but the training processes were not described.	Yes	In Figure 2, the VAE-Deconv component was absent. Within Figure 3, the depiction of outcomes is partial across various methods, and the rationale for excluding specific subfigures has not been presented. The evaluation of privacy remains either unaddressed or, at minimum, the outcomes pertaining to the alternative comparative methods and EVA_c were absent from the presentation.
Gootjes-Dreesbach et al. [31]			Yes	Comparative analyses between the actual patients and virtual patients were only shown for the PPMI dataset. In Figure 6, the depiction of decoded real patients was missing from the subset pertaining to SP513.
Sood et al. [80]			Yes	Comparisons between synthetic and original variables were selectively delineated for a subset of the variables under consideration.

Authors	Performance bias	Explanation	Reporting bias	Explanation
Sood et al. [81]			Yes	Comparisons between synthetic and original variables were selectively delineated for a subset of the variables under consideration.
Fisher et al. [27]			Yes	The authors had decided to confine the outcome section to a subset of data characterized as partially synthetic. Notably, some of the evaluation techniques could have been suitably extended to encompass fully synthetic data. The rationale behind this decision remains unclear.
El Kababji et al. [42]	Possibly	The method was compared to others, and the authors appropriately noted that some methods had limited capabilities for hyperparameter tuning. While this is a valuable observation, it is possible that these limitations could have influenced the results, potentially introducing performance bias.		
Yoon et al. [100]	Possibly	The method was compared to other methods, but the training processes were not described.		
Kuo et al. [47]			Yes	Some alternative GAN training methods were also tested, but their results were not displayed.
Hashemi et al. [37]	Possibly	The method was compared to other methods, but the training processes were not described.		
Sun, Lin & Yan [83]	Possibly	The method was compared to other methods, but the training processes were not described.	Yes	The utility plot (Figure 4) was presented only for the primary method MSIC. The privacy plot (Figure 5) omitted certain methods (LSTM+MLP, MTGAN), and the gram probability plots also excluded some methods (LSTM+MLP, MTGAN, Med(B/W)GAN).
Wang & Sun [94]			Yes	Certain results were excluded regarding the comparison methods, such as privacy and utility (prediction) metrics.
Das, Wang & Sun [20]			Yes	The dimension-wise probabilities for lab tests were excluded, and the patient-wise correlation figures (Figures 4 and 5) for the other methods were not shown. Additionally, Figures 6 and 9, along with Table 3, miss some of the reference methods.
Raab, Nowok & Dibben [72]			Yes	Analyses concerning the marginal distributions and the preservation of temporal correlations of discrete variables were not presented.
Haleem et al. [33]	Possibly	The method was compared to other methods, but the training processes were not described.	Possibly	From the provided figures on the original data, it seems that comparison results were shown for only a subset of the variables in the dataset. However, this cannot be confirmed due to the lack of a detailed description of the original data.
Pang et al. [68]	Possibly	The method was compared to other methods, but the training processes were not described.	Yes	Certain measurements (privacy metrics) and figures (4, 6, 7) were not provided for the comparison methods.
Kühnel et al. [46]			Yes	Some univariate comparisons were missing; specifically, Table 2 did not include all variables, and this information was also absent from the supplemental material.

A.4.3 Individual study reporting quality assessment (detailed explanations)

Detailed explanations of the identified reporting quality deviations within each study are presented in the table below. **Inconsistency of reporting** refers to utilization of 1) identical terminology or notations to signify distinct phenomena and lacking clarification (e.g., using "noise" for both original data variation and additional privacy mechanism-induced noise without clear differentiation) or 2) disparate notations to represent the same phenomenon, both 1 and 2 introduce a potential risk of misunderstanding. **Imprecision of reporting** refers to the lack of precision (e.g. p-values reported with varying accuracies) or clarity in the presentation of information, which may lead to ambiguity or difficulty in understanding the reported data. **Indirectness of reporting** involves conveying information in a manner that is not straightforward or explicit, albeit to a lesser extent than observed in reporting bias, potentially requiring the reader to infer or deduce certain details. This can introduce a level of uncertainty or make the interpretation less direct.

Authors	Inconsistency	Explanation	Imprecision	Explanation	Indirectness	Explanation
Belgodere et al. [8]					Yes	The exact number of variables was not clearly specified; instead, the term "about 18" was used (p. 9, right column, second-to-last paragraph)
Bhanot et al. [10]			Yes	The number of patients was not reported precisely ("The data set has over 30 K records", page 2).		

Authors	Inconsistency	Explanation	Imprecision	Explanation	Indirectness	Explanation
Biswal et al. [11]			Yes	The number of clinicians used to evaluate the realism score was not reported. Information on the minimum and maximum number of visits per patient and the minimum number of codes per visit was not given.	Yes	Full details about the presence disclosure test were not provided. Abbreviations like ELBO were unspecified. The nature of preliminary evaluations mentioned in the appendix remained unclear.
Das, Wang & Sun [20]					Yes	In equations (11) and (12), it was unclear how the numerator and denominator are derived from the data.
Gootjes-Dreesbach et al. [31]	Yes	Utilized three distinct notations for the differential privacy budget parameter.	Yes	Subfigure 9.1 did not specify the epsilon used in that figure.		
Li et al. [54]			Yes	Statistically significant p-values were not reported as precisely as values above 0.05.	Yes	The meaning of mean and standard deviation for a discrete-valued feature was unclear (page 13, section 4.3).<br>Figure 5 states that the y-axis represented the probability distribution of Mechanical Ventilation and Vasopressor being applied ("On"). It was unclear how the y-axis can exceed the range of [0,1].<br>In reference to differential privacy, it was stated that delta ≤ 0.001 (p. 21), but it was unclear what the exact delta was in each situation, e.g. if the delta remained constant for all values of epsilon presented in Figure 7b.
Sood et al. [80]			Yes	The number and types of variables employed in the actual synthesis of data remained unclear.		
Sun, Lin & Yan [83]					Yes	It is unclear how the Dimwise score was computed. Additionally, the model used to assess utility wasn't specified, leaving out important details about the task and the exact model applied.
Wendland et al. [95]			Yes	The p-value on page 3, right column, first paragraph, was reported with different precision compared to the subsequent p-values (which have two significant figures).		
Yoon et al. [100]					Yes	Some abbreviations, like AP, were either not explained or not introduced when first mentioned.
Zhang et al. [105]					Yes	The data description in Table 1 shows the gender distribution, but the article lacks clarity on whether this variable was utilized in data synthesis or analyses.

A.5 Study selection: examples of excluded publications

The primary reason for exclusion was wrong data type (n = 237), mostly cross-sectional, e.g., [1, 69, 89, 104], survival [13, 24, 43, 98] or time-series data [39, 87, 92]. Publications compromising the temporal structure in longitudinal data were categorized as having wrong data type, e.g., [5, 21, 55]. Publications lacking SDG (n = 92) were typically introductions of a specific synthetic data framework, e.g., [12, 23, 57, 60] or data simulations [28, 56, 61]. Exclusions due to partially synthetic data (n = 51) were largely related to data augmentation using techniques such as Synthetic Minority Over-Sampling Technique (SMOTE) [50] or its variants, e.g., [59, 70, 78, 84, 91].

We excluded 38 publications as we could not determine their eligibility, stemming from incomplete data, incomplete method description, or restricted access to the cited references, data, or algorithms, e.g., [30, 41, 82, 103]. Additionally, 32 studies were excluded for relying solely on standard probability distributions to simulate data, e.g., [22, 38, 44, 66]. Furthermore, 20 studies were excluded for failing to acknowledge the longitudinal nature of data, e.g., [3, 4, 19, 26, 34, 53], although the original datasets included variables with repeated measurements. Lastly, we identified four duplicates and one publication of wrong literature type (thesis).

A.6 Reference methods

The following table presents the reference methods used to benchmark the primary methods. Those reference methods included in the main review as primary methods are marked with "(included)" in the last column. Some reference methods were not included as primary methods because their original publications did not design or implement the method for longitudinal data, or we could not confirm how the authors modified the method to address the longitudinal aspect. Therefore, we excluded these methods from further inspection but included them here, as they may still be valuable for other users.

Study	Primary method	Reference methods
Biswal et al. [11]	EVA	EVAc
		biLSTM [76]
		VAE-LSTM [14]
		VAE-Deconv [77]
Das, Wang & Sun [20]	TWIN	KNN
		EVA [11] (included)
		SynTEG [105] (included)
		PromptEHR [94] (included)
Haleem et al. [33]	TC-MultiGAN	CTGAN [97] (included)
		Gaussian Copula [50]
		Dragan GAN [45]
		Cramer GAN [9]
Hashemi et al. [37]	TAP-GAN	TimeGAN [99]
Kühnel et al. [46]	VAMBN-MT	VAMBN [31] (included)
		VAMBN-FT
Kuo et al. [47]	HGG+VAE+Buffer	Health Gym GAN (HGG) [49] (included)
		HGG+G_EOT+VAE+Buffer
Li at al. [54]	EHR-M-GAN	C-RNN-GAN [62]
		RCGAN [25]
		TimeGAN [99]
		MedGAN [17]
		seqGAN[101]
		SynTEG [105] (included)
		DualAEE [51]
		PrivBayes [104]
Lu et al. [58]	MTGAN	MedGAN [17]
		CTGAN [97] (included)
		EMR-WGAN [107]
		RDP-CGAN [88]
		WGAN-GP [32]
		TimeGAN [99]
		T-CGAN [75]
Pang et al. [68]	CEHR-GPT	CEHR-BERT
		GPT-Vanilla
		GPT-OUTPAT
Sun, Lin & Yan [83]	MSIC	MedGAN [7]
		Med(B/W)GAN [5]
		EVA [11] (included)
		MTGAN [58] (included)
		PromptEHR [94] (included)
Theodorou et al. [86]	HALO	HALO-coarse
		EVA [11] (included)
		SynTEG [105] (included)
		LSTM [50]

Study	Primary method	Reference methods
		GPT-2 [73]
Theodorou et al. [85]	ConSequence	HALO [86] (included)<br>Semantic Loss [96]<br>CCN [29]<br>MultiPlexNet [40]<br>SPL [2]
Wang & Sun [94]	PromptEHR	LSTM + MLP<br>LSTM + MedGAN [17]<br>SynTEG [105] (included)<br>GPT-2 [73]
Wendland et al. [95]	MultiNODEs	VAMBN [31] (included)
Yoon et al. [100]	EHR-Safe	TimeGAN [99]<br>RCGAN[25]<br>C-RNN-GAN [62]
Yu, He & Raghunathan [102]	SPMI	IVEware Version 0.3 [74]<br>Synthpop [65] (included)

A.7 Datasets used in the included publications

The following table lists all datasets used in the included publications to generate synthetic data.

Datasets	Data type	Availability	Study	Subjects	Numerical variables	Categorical variables	Time-varying variables	Repeated measurements
MIMIC-III	Clinical database	Public	[7]	8 260	9	1	9	5
			[54]	28 344	78	20	98	24
			[58]	7 493	0	4 880	4 880	avg. 2.6
			[48,49]	3 910	9	13	20	48
			[49]	2 164	35	11	42	2–20
			[86]	929 268	0	9882	9882	avg. 3.9
			[86]	46 520	15	31	17	avg. 11.9
			[94]	46 520	0	4	4	NA
			[83]	5 794	0	3	3	avg. 2.1
			[8]	14 681	NA	NA	~18	48
			[85]	46 520	0	1616	1610	avg. 1.3
			[100]	19 946	79	11	84	1–30
MIMIC-IV	Clinical database	Public	[58]	10 000	0	6 102	6 102	avg. 3.6
			[37]	9 133	6	0	6	5
			[83]	36 615	0	3	3	avg. 3.5
			[64]	6 535	NA	3	3	NA
PPMI	Patient data	Public	[80]	362	NA	NA	38	2–12
			[95]	354	53	15	25	5–12
			[31]	557	NA	NA	38*	5
eICU	Clinical database	Public	[54]	99 015	55	19	74	24
			[100]	198 707	53	1	51	2–50
VUMC	EHR data	No	[106]	59 617	0	1 276	1 276	25–200
	Synthetic derivate	No	[105]	2 187 629	0	1 799	1 799	avg.12.1
ADNI	Patient data	Public	[80]	689	NA	NA	18	4
Alberta Health	EHR data	Upon request	[63]	100 000	NA	NA	6	max. 1000
All of Us	EHR data	Public	[106]	59 617	0	526	526	10–200
ASD	Health data	No	[10]	> 280 000	7	2	7	10
ATUS	Behavioral data	Public	[10]	> 30 000	1	4	1	30
Breast cancer	Clinical trial data	Upon request	[20]	971	0	3	3	max. 14
CCTG MA27	Clinical trial data	No	[42]	7 576	NA	NA	NA	NA
CDC	EHR data	No	[93]	9 298	1	100	88	2–11
CODR-AD	Clinical database	No	[27]	1 909	38	6	36	7
CUIMC New York Presbyterian	EHR data	No	[68]	~ 2 300 000	3	8	5	2–102
DONALD	Health data	No	[46]	1312	28	7	33	16
GATEKEEPER	Health data	No	[33]	86	NA	NA	NA	max. 24
HiRID	Clinical database	Public	[54]	14 129	50	39	89	24

Datasets	Data type	Availability	Study	Subjects	Numerical variables	Categorical variables	Time-varying variables	Repeated measurements
HIV	EuResist integrated database	Public	[47–49]	8 916	3	12	13	10–100
HRS	Longitudinal survey	No	[102]	12 652	7	41	11	2–3
Multi-census	Census data	No	[90]	NA	NA	NA	NA	NA
NACC	Patient data	Public	[95]	2 284	4	3	3	4
NSABP B34	Clinical trial data	No	[42]	3 323	NA	NA	NA	NA
PAMF EHR	EHR data	No	[11]	258 555	0	10 437	10 437	avg. 53.8
REaCT	Clinical trial data	No	[42]	48–230	NA	NA	NA	NA
SIPP	Longitudinal survey	Yes	[15]	23 374	0	1	1	12
Small cell lung carcinoma	Clinical trial data	Upon request	[20]	77	0	3	3	max. 5
SP513	Clinical trial data	No	[31]	560	NA	NA	35*	2–11*
SPRINT	Clinical trial data	No	[7]	6 502	3	1	3	12
Status File	Employment data	No	[6]	3 511 824	5	24	22	24
SWOG 0307	Clinical trial data	No	[42]	6 097	NA	NA	NA	NA
TREND	Patient data	No	[81]	1 178	1	18	18	4
UK LS	Census data	No	[72]	> 186 000	1	4	5	2
Unnamed	Patient data	No	[79]	580 000	NA	NA	2	2
US claims data	Insurance data	No	[85]	1 006 321	0	1824	1817	avg. 35.4

NA: not available; EHR: electronic health records; avg.: average; max.: maximum; ~: approximately; *: calculated from presented materials by the corresponding author of this systematic literature review

A.8 Models used to evaluate resemblance and utility

The following list outlines how different models were used to assess the resemblance or utility of synthetic data (related to Section 3.5 in the main text). An asterisk placed after a reference indicates that, in the model-based assessment, the longitudinal structure of the data was either ignored by the method, or its acknowledgment could not be determined from the record; otherwise, it is explained how the longitudinal structure was accounted for.

- Resemblance (multivariate): *Random forest* is a supervised method that uses an ensemble of decision trees to conduct prediction or classification. [80]* take data containing both real and synthetic patients and train a random forest to separate the two classes, using the real/synthetic status as a label and all other variables as explanatory. A badly performing classification is an indicator of good resemblance.
- Resemblance (multivariate): [106]* use three strategies (naïve, transfer learning, and fine-tuning) to train a discriminator based on Jensen-Shannon divergences to carry out a resemblance evaluation in the same manner as [80] with the random forest above.
- Resemblance (multivariate): *Factor analysis* is a classical method for decomposing variation into a small set of joint factors and variable-specific error terms. [102]* compare the loadings estimated with factor analysis from real and synthetic datasets to assess their level of resemblance.
- Resemblance (multivariate): *Support vector machine* (SVM) is a supervised learning algorithm that classifies data by finding the optimal boundary (hyperplane) that best separates different classes. [64] trained an SVM classifier to learn to distinguish between real and synthetic trajectories. Low classification performance indicates good resemblance.
- Resemblance (temporal preservation): *Recurrent Neural Network* (RNN) is a type of artificial neural network designed to handle sequential data. Unlike regular neural networks, which process inputs independently, RNNs keep track of previous inputs through a hidden "memory" state that is updated step by step, allowing utilization of temporal information. [37] applied RNN to data in long format to conduct one-step ahead predictions. Closely matching prediction errors between real and synthetic data are indicative of good temporal preservation.
- Utility (inference): *Linear regression* is a statistical method used to estimate the relationship between a dependent variable and one or more independent variables by fitting a linear function. Both [6] and [102] compared the estimates of linear regression coefficients between real and synthetic data. The former inspected linear regression on yearly measurements with

time as the predictor, while the latter modeled change between two time points by introducing a new variable representing the difference between measurements; in addition, they examined the corresponding standard errors.

- Utility (inference): *Logistic regression* is a statistical method for predicting the values of a binary variable based on a linear combination of a set of explanatory variables. [72]* and [102]* evaluated inferential utility by comparing logistic regression coefficients and their standard errors between real and synthetic data.
- Utility (inference): [79]* used four different models (doubly robust, the propensity score stratification, the propensity matching, and the inverse probability treatment weighting) to estimate causal effects from synthetic data.
- Utility (inference): *Mixed effect models* are statistical regression models that allow the presence of correlation between individual observations, making them naturally suited for modelling longitudinal data with repeated measurements. They also naturally account for unbalancedness in the data. [46] use mixed effect models to estimate and compare temporal trends between real and synthetic data.
- Utility (inference): *Generalized estimation equations* (GEEs) are closely related to mixed effect models and similarly permit the correlation between individual observations, while at the same time offering additional flexibility in the choice of the mean and variance structure. Using the synthetic data, [42] repeated the original analyses performed on real data in order to estimate the risk difference between the two treatment arms across chemotherapy cycles.
- Utility (prediction): [7, 8, 68, 100]* applied logistic regression to predict binary outcomes (e.g., the presence of a disease or condition) using all or a subset of available variables, including repeated measurements, as input (explanatory) variables.
- Utility (prediction): [7, 81, 95,100]* used random forest to predict binary outcome variables in a similar manner as logistic regression mentioned above.
- Utility (prediction): *Long Short-Term Memory* (LSTM) network is a type of RNN designed to learn long-range dependencies in sequential data by using memory cells and gating mechanisms to control the flow of information. [20,54, 86]* used LSTM to predict a binary outcome, such as, death, adverse event, intervention or occurrence of a disease. [94]* used LSTM for multilabel prediction and [11] predicted future conditions using the previous conditions as input.
- Utility (prediction): [7]* used SVM to classify treatment arms, and [64]* used SVM to classify outliers.
- Utility (prediction): *Batch-constrained Q-learning* is a reinforcement learning method for learning decision rules from a fixed dataset, where the generative model algorithm only considers actions that actually appear in the data, to prevent unreliable predictions about actions it has not seen. [49]* used the method to predict discrete clinical actions based on patient status and compared the resulting frequencies of predicted actions between the models trained on real and synthetic data.
- Utility (prediction): *Multilayer perceptron* is a feedforward neural network made up of multiple layers of interconnected nodes that can model complex nonlinear relationships. [20] trained an MLP to predict the next time step's adverse events taking previous visits as the input. [64] trained an MLP to predict heart failure based on the previous encounters.
- Utility (prediction): *Gradient boosting tree ensemble* is a machine learning method that builds a series of decision trees, where each new tree corrects the errors of the previous ones, and combines them into a predictive model. [100]* used the model for mortality prediction (binary outcome).
- Utility (prediction): *Gated recurrent unit* is a type of recurrent neural network that uses gating mechanisms to efficiently capture dependencies in sequential data. [100]* used the model for mortality prediction (binary outcome).

A.9 PRISMA checklist

PRISMA 2020 Checklist

Section and Topic	Item #	Checklist item	Location where item is reported
**TITLE**			
Title	1	Identify the report as a systematic review.	Title
**ABSTRACT**			
Abstract	2	See the PRISMA 2020 for Abstracts checklist.	Abstract
**INTRODUCTION**			
Rationale	3	Describe the rationale for the review in the context of existing knowledge.	Section 1.1
Objectives	4	Provide an explicit statement of the objective(s) or question(s) the review addresses.	Section 1.2
**METHODS**			
Eligibility criteria	5	Specify the inclusion and exclusion criteria for the review and how studies were grouped for the syntheses.	Section 2.1
Information sources	6	Specify all databases, registers, websites, organisations, reference lists and other sources searched or consulted to identify studies. Specify the date when each source was last searched or consulted.	Section 2.2
Search strategy	7	Present the full search strategies for all databases, registers and websites, including any filters and limits used.	Section 2.3 + A.1
Selection process	8	Specify the methods used to decide whether a study met the inclusion criteria of the review, including how many reviewers screened each record and each report retrieved, whether they worked independently, and if applicable, details of automation tools used in the process.	Section 2.4 + A.2
Data collection process	9	Specify the methods used to collect data from reports, including how many reviewers collected data from each report, whether they worked independently, any processes for obtaining or confirming data from study investigators, and if applicable, details of automation tools used in the process.	Section 2.5
Data items	10a	List and define all outcomes for which data were sought. Specify whether all results that were compatible with each outcome domain in each study were sought (e.g. for all measures, time points, analyses), and if not, the methods used to decide which results to collect.	Section 2.5 + A.3
	10b	List and define all other variables for which data were sought (e.g. participant and intervention characteristics, funding sources). Describe any assumptions made about any missing or unclear information.	Section 2.5 + A.3
Study risk of bias assessment	11	Specify the methods used to assess risk of bias in the included studies, including details of the tool(s) used, how many reviewers assessed each study and whether they worked independently, and if applicable, details of automation tools used in the process.	Section 2.6. + A.4
Effect measures	12	Specify for each outcome the effect measure(s) (e.g. risk ratio, mean difference) used in the synthesis or presentation of results.	Not applicable
Synthesis methods	13a	Describe the processes used to decide which studies were eligible for each synthesis (e.g. tabulating the study intervention characteristics and comparing against the planned groups for each synthesis (item #5)).	Section 2.7
	13b	Describe any methods required to prepare the data for presentation or synthesis, such as handling of missing summary statistics, or data conversions.	Section 2.5<br>Section 2.7
	13c	Describe any methods used to tabulate or visually display results of individual studies and syntheses.	Section 2.7
	13d	Describe any methods used to synthesize results and provide a rationale for the choice(s). If meta-analysis was performed, describe the model(s), method(s) to identify the presence and extent of statistical heterogeneity, and software package(s) used.	Section 2.7
	13e	Describe any methods used to explore possible causes of heterogeneity among study results (e.g. subgroup analysis, meta-regression).	Not applicable
	13f	Describe any sensitivity analyses conducted to assess robustness of the synthesized results.	Not applicable
Reporting bias assessment	14	Describe any methods used to assess risk of bias due to missing results in a synthesis (arising from reporting biases).	Not applicable
Certainty assessment	15	Describe any methods used to assess certainty (or confidence) in the body of evidence for an outcome.	Not applicable

PRISMA 2020 Checklist

Section and Topic	Item #	Checklist item	Location where item is reported
**RESULTS**			
Study selection	16a	Describe the results of the search and selection process, from the number of records identified in the search to the number of studies included in the review, ideally using a flow diagram.	Section 3.1
	16b	Cite studies that might appear to meet the inclusion criteria, but which were excluded, and explain why they were excluded.	A.4
Study characteristics	17	Cite each included study and present its characteristics.	Section 3.2
Risk of bias in studies	18	Present assessments of risk of bias for each included study.	Section 3.3 + A.4
Results of individual studies	19	For all outcomes, present, for each study: (a) summary statistics for each group (where appropriate) and (b) an effect estimate and its precision (e.g. confidence/credible interval), ideally using structured tables or plots.	Not applicable
Results of syntheses	20a	For each synthesis, briefly summarise the characteristics and risk of bias among contributing studies.	Section 3 + 4
	20b	Present results of all statistical syntheses conducted. If meta-analysis was done, present for each the summary estimate and its precision (e.g. confidence/credible interval) and measures of statistical heterogeneity. If comparing groups, describe the direction of the effect.	Section 3.4<br>Section 3.5
	20c	Present results of all investigations of possible causes of heterogeneity among study results.	Section 4
	20d	Present results of all sensitivity analyses conducted to assess the robustness of the synthesized results.	Not applicable
Reporting biases	21	Present assessments of risk of bias due to missing results (arising from reporting biases) for each synthesis assessed.	Not applicable
Certainty of evidence	22	Present assessments of certainty (or confidence) in the body of evidence for each outcome assessed.	Section 4
**DISCUSSION**			
Discussion	23a	Provide a general interpretation of the results in the context of other evidence.	Section 4
	23b	Discuss any limitations of the evidence included in the review.	Section 4.1
	23c	Discuss any limitations of the review processes used.	Section 4.1
	23d	Discuss implications of the results for practice, policy, and future research.	Section 4.2
**OTHER INFORMATION**			
Registration and protocol	24a	Provide registration information for the review, including register name and registration number, or state that the review was not registered.	Acknowledgements
	24b	Indicate where the review protocol can be accessed, or state that a protocol was not prepared.	Acknowledgements
	24c	Describe and explain any amendments to information provided at registration or in the protocol.	Acknowledgements
Support	25	Describe sources of financial or non-financial support for the review, and the role of the funders or sponsors in the review.	Acknowledgements
Competing interests	26	Declare any competing interests of review authors.	Title page; no COI
Availability of data, code and other materials	27	Report which of the following are publicly available and where they can be found: template data collection forms; data extracted from included studies; data used for all analyses; analytic code; any other materials used in the review.	Acknowledgements

From: Page MJ, McKenzie JE, Bossuyt PM, Boutron I, Hoffmann TC, Mulrow CD, et al. The PRISMA 2020 statement: an updated guideline for reporting systematic reviews. BMJ 2021;372:n71. doi: 10.1136/bmj.n71

For more information, visit: http://www.prisma-statement.org/

APPENDIX REFERENCES

[1] Nazmiye Ceren Abay, Yan Zhou, Murat Kantarcioglu, Bhavani Thuraisingham, and Latanya Sweeney. 2019. Privacy Preserving Synthetic Data Release Using Deep Learning. In Lecture Notes in Computer Science. Springer Verlag, 510–526. https://doi.org/10.1007/978-3-030-10925-7_31

[2] Kareem Ahmed, Stefano Teso, Kai Wei Chang, Guy Van den Broeck, and Antonio Vergari. 2022. Semantic Probabilistic Layers for Neuro-Symbolic Learning. In Advances in Neural Information Processing Systems, 2022. https://dl.acm.org/doi/10.5555/3600270.3602441

[3] Clinton J. Andrews, Maryann Sorensen Allacci, Jennifer Senick, Handi Chandra Putra, and Ioanna Tsoulou. 2016. Using synthetic population data for prospective modeling of occupant behavior during design. Energy Build 126, (August 2016), 415–423. https://doi.org/10.1016/j.enbuild.2016.05.049

[4] Max Baak, Simon Brugman, Lorraine D'almeida, Ilan Fridman Rojas, and Jean-Baptiste Oger. 2022. Synthsonic: Fast, Probabilistic modeling and Synthesis of Tabular Data. In Proceedings of the 25th International Conference on Artificial Intelligence and Statistics, 2022, PMLR 151:4747-4763. Retrieved from https://proceedings.mlr.press/v151/baak22a.html

[5] Mrinal Kanti Baowaly, Chia Ching Lin, Chao Lin Liu, and Kuan Ta Chen. 2019. Synthesizing electronic health records using improved generative adversarial networks. Journal of the American Medical Informatics Association 26, 3 (March 2019), 228–241. https://doi.org/10.1093/jamia/ocy142

[6] Andrés F. Barrientos, Alexander Bolton, Tom Balmat, Jerome P. Reiter, John M. de Figueiredo, Ashwin Machanavajjhala, Yan Chen, Charley Kneifel, and Mark Delong. 2018. Providing access to confidential research data through synthesis and verification: An application to data on employees of the U.S. federal government. Annals of Applied Statistics 12, 2 (June 2018), 1124–1156. https://doi.org/10.1214/18-AOAS1194

[7] Brett K. Beaulieu-Jones, Zhiwei Steven Wu, Chris Williams, Ran Lee, Sanjeev P. Bhavnani, James Brian Byrd, and Casey S. Greene. 2019. Privacy-Preserving Generative Deep Neural Networks Support Clinical Data Sharing. Circ Cardiovasc Qual Outcomes 12, 7 (July 2019), e005122. https://doi.org/10.1161/CIRCOUTCOMES.118.005122

[8] Brian Belgodere, Pierre Dognin, Adam Ivankay, Igor Melnyk, Youssef Mroueh, Aleksandra Mojsilovic, Jiri Navratil, Apoorva Nitsure, Inkit Padhi, Mattia Rigotti, Jerret Ross, Yair Schiff, Radhika Vedpathak, and Richard A. Young. 2023. Auditing and Generating Synthetic Data with Controllable Trust Trade-offs. arXiv preprint (April 2023). https://doi.org/10.48550/arXiv.2304.10819

[9] Marc G. Bellemare, Ivo Danihelka, Will Dabney, Shakir Mohamed, Balaji Lakshminarayanan, Stephan Hoyer, and Rémi Munos. 2017. The Cramer Distance as a Solution to Biased Wasserstein Gradients. arXiv preprint (May 2017). https://doi.org/10.48550/arXiv.1705.10743

[10] Karan Bhanot, Joseph Pedersen, Isabelle Guyon, and Kristin P. Bennett. 2022. Investigating synthetic medical time-series resemblance. Neurocomputing 494, (July 2022), 368–378. https://doi.org/10.1016/j.neucom.2022.04.097

[11] Siddharth Biswal, Soumya Ghosh, Jon Duke, Bradley Malin, Walter Stewart, and Jimeng Sun. 2021. EVA: Generating Longitudinal Electronic Health Records Using Conditional Variational Autoencoders. In Proceedings of the 6th Machine Learning for Healthcare Conference, August 06, 2021. PMLR, 260–282. Retrieved from https://proceedings.mlr.press/v149/biswal21a.html

[12] March Boedihardjo, Thomas Strohmer, and Roman Vershynin. 2023. Privacy of Synthetic Data: A Statistical Framework. IEEE Trans Inf Theory 69, 1 (January 2023), 520–527. https://doi.org/10.1109/TIT.2022.3216793

[13] Federico Bonofiglio, Martin Schumacher, and Harald Binder. 2020. Recovery of original individual person data (IPD) inferences from empirical IPD summaries only: Applications to distributed computing under disclosure constraints. Stat Med 39, 8 (April 2020), 1183–1198. https://doi.org/10.1002/sim.8470

[14] Samuel R. Bowman, Luke Vilnis, Oriol Vinyals, Andrew M. Dai, Rafal Jozefowicz, and Samy Bengio. 2016. Generating sentences from a continuous space. In CoNLL 2016 - 20th SIGNLL Conference on Computational Natural Language Learning, Proceedings, 2016. https://doi.org/10.18653/v1/k16-1002

[15] Mark Bun, Marco Gaboardi, Marcel Neunhoeffer, and Wanrong Zhang. 2024. Continual Release of Differentially Private Synthetic Data from Longitudinal Data Collections. Proceedings of the ACM on Management of Data 2, 2 (May 2024), 1–26. https://doi.org/10.1145/3651595

[16] N. V. Chawla, K. W. Bowyer, L. O. Hall, and W. P. Kegelmeyer. 2002. SMOTE: Synthetic Minority Over-sampling Technique. Journal of Artificial Intelligence Research 16, (June 2002), 321–357. https://doi.org/10.1613/jair.953

[17] Edward Choi, Siddharth Biswal, Bradley Malin, Jon Duke, Walter F Stewart, and Jimeng Sun. 2017. Generating Multi-label Discrete Patient Records using Generative Adversarial Networks. In Proceedings of the 2nd Machine Learning for Healthcare Conference, August 2017. PMLR, Boston, Massachusetts, 286–305. Retrieved from https://proceedings.mlr.press/v68/choi17a.html

[18] arXiv.org submitters, arXiv Dataset, Kaggle; 2024. https://doi.org/10.34740/KAGGLE/DSV/7548853.

[19] Fida K. Dankar and Mahmoud Ibrahim. 2021. Fake It Till You Make It: Guidelines for Effective Synthetic Data Generation. Applied Sciences 11, 5 (February 2021), 2158. https://doi.org/10.3390/app11052158

[20] Trisha Das, Zifeng Wang, and Jimeng Sun. 2023. TWIN: Personalized Clinical Trial Digital Twin Generation. In Proceedings of the 29th ACM SIGKDD Conference on Knowledge Discovery and Data Mining, August 06, 2023. ACM, New York, NY, USA, 402–413. https://doi.org/10.1145/3580305.3599534

[21] Saloni Dash, Ritik Dutta, Isabelle Guyon, Adrien Pavao, Andrew Yale, and Kristin P. Bennett. 2019. Synthetic Event Time Series Health Data Generation. arXiv preprint (November 2019). https://doi.org/10.48550/arXiv.1911.06411

[22] Hakan Demirtas and Yasemin Yavuz. 2015. Concurrent Generation of Ordinal and Normal Data. J Biopharm Stat 25, 4 (July 2015), 635–650. https://doi.org/10.1080/10543406.2014.920868

[23] Kudakwashe Dube and Thomas Gallagher. 2014. Approach and Method for Generating Realistic Synthetic Electronic Healthcare Records for Secondary Use. In Lecture Notes in Computer Science (including subseries Lecture Notes in Artificial Intelligence and Lecture Notes in Bioinformatics). 69–86. https://doi.org/10.1007/978-3-642-53956-5_6

[24] Khaled El Emam, Lucy Mosquera, and Chaoyi Zheng. 2021. Optimizing the synthesis of clinical trial data using sequential trees. Journal of the American Medical Informatics Association 28, 1 (January 2021), 3–13. https://doi.org/10.1093/jamia/ocaa249

[25] Cristóbal Esteban, Stephanie L. Hyland, and Gunnar Rätsch. 2017. Real-valued (Medical) Time Series Generation with Recurrent Conditional GANs. arXiv prerint (June 2017). https://doi.org/10.48550/arXiv.1706.02633

[26] Joseph Feldman and Daniel R. Kowal. 2022. Bayesian data synthesis and the utility-risk trade-off for mixed epidemiological data. Ann Appl Stat 16, 4 (December 2022), 2577–2602. https://doi.org/10.1214/22-AOAS1604

[27] Charles K. Fisher, Aaron M. Smith, Jonathan R. Walsh, Adam J. Simon, Chris Edgar, Clifford R. Jack, David Holtzman, David Russell, Derek Hill, Donald Grosset, Fred Wood, Hugo Vanderstichele, John Morris, Kaj Blennow, Ken Marek, Leslie M Shaw, Marilyn Albert, Michael Weiner, Nick Fox, Paul Aisen, Patricia E. Cole, Ronald Petersen, Todd Sherer, and Wayne Kubick. 2019. Machine learning for comprehensive forecasting of Alzheimer's Disease progression. Sci Rep 9, 1 (September 2019), 13622. https://doi.org/10.1038/s41598-019-49656-2

[28] Laurie A. Garrow, Tudor D. Bodea, and Misuk Lee. 2010. Generation of synthetic datasets for discrete choice analysis. Transportation (Amst) 37, 2 (March 2010), 183–202. https://doi.org/10.1007/s11116-009-9228-6

[29] Eleonora Giunchiglia and Thomas Lukasiewicz. 2021. Multi-Label Classification Neural Networks with Hard Logical Constraints. Journal of Artificial Intelligence Research 72, (2021). https://doi.org/10.1613/JAIR.1.12850

[30] Andre Goncalves, Priyadip Ray, Braden Soper, Jennifer Stevens, Linda Coyle, and Ana Paula Sales. 2020. Generation and evaluation of synthetic patient data. BMC Med Res Methodol 20, 1 (December 2020), 108. https://doi.org/10.1186/s12874-020-00977-1

[31] Luise Gootjes-Dreesbach, Meemansa Sood, Akrishta Sahay, Martin Hofmann-Apitius, and Holger Fröhlich. 2020. Variational Autoencoder Modular Bayesian Networks for Simulation of Heterogeneous Clinical Study Data. Front Big Data 3, (May 2020). https:// doi.org/10.3389/fdata.2020.00016

[32] Ishaan Gulrajani, Faruk Ahmed, Martin Arjovsky, Vincent Dumoulin, and Aaron Courville. 2017. Improved training of wasserstein GANs. In Advances in Neural Information Processing Systems, 2017. https://dl.acm.org/doi/10.5555/3295222.3295327

[33] Muhammad Salman Haleem, Audrey Ekuban, Alessio Antonini, Silvio Pagliara, Leandro Pecchia, and Carlo Allocca. 2023. Deep-Learning-Driven Techniques for Real-Time Multimodal Health and Physical Data Synthesis. Electronics (Switzerland) 12, 9 (May 2023). https://doi.org/10.3390/electronics12091989

[34] Frederik Harder, Kamil Adamczewski, and Mijung Park. 2021. DP-MERF: Differentially Private Mean Embeddings with Random Features for Practical Privacy-Preserving Data Generation. arXiv preprint (2021). https://doi.org/10.48550/arXiv.2002.11603

[35] Paul A. Harris, Robert Taylor, Brenda L. Minor, Veida Elliott, Michelle Fernandez, Lindsay O'Neal, Laura McLeod, Giovanni Delacqua, Francesco Delacqua, Jacqueline Kirby, and Stephany N. Duda. 2019. The REDCap consortium: Building an international community of software platform partners. J Biomed Inform 95, (July 2019), 103208. https://doi.org/10.1016/j.jbi.2019.103208

[36] Paul A. Harris, Robert Taylor, Robert Thielke, Jonathon Payne, Nathaniel Gonzalez, and Jose G. Conde. 2009. Research electronic data capture (REDCap)—A metadata-driven methodology and workflow process for providing translational research informatics support. J Biomed Inform 42, 2 (April 2009), 377–381. https://doi.org/10.1016/j.jbi.2008.08.010

[37] Atiye Sadat Hashemi, Kobra Etminani, Amira Soliman, Omar Hamed, and Jens Lundström. 2023. Time-series Anonymization of Tabular Health Data using Generative Adversarial Network. In 2023 International Joint Conference on Neural Networks (IJCNN), June 18, 2023. IEEE, 1–8. https://doi.org/10.1109/IJCNN54540.2023.10191367

[38] Sven Helfer, Michéle Kümmel, Franziska Bathelt, and Martin Sedlmayr. 2021. Generating Enriched Synthetic German Hospital Claims Data – A Use Case Driven Approach. In German Medical Data Sciences: Bringing Data to Life. 58–65. https://doi.org/10.3233/SHTI210051

[39] Mikel Hernandez, Gorka Epelde, Andoni Beristain, Roberto Álvarez, Cristina Molina, Xabat Larrea, Ane Alberdi, Michalis Timoleon, Panagiotis Bamidis, and Evdokimos Konstantinidis. 2022. Incorporation of Synthetic Data Generation Techniques within a Controlled Data Processing Workflow in the Health and Wellbeing Domain. Electronics (Basel) 11, 5 (March 2022), 812. https://doi.org/10.3390/electronics11050812

[40] Nicholas Hoernle, Rafael Michael Karampatsis, Vaishak Belle, and Kobi Gal. 2022. MultiplexNet: Towards Fully Satisfied Logical Constraints in Neural Networks. In Proceedings of the 36th AAAI Conference on Artificial Intelligence, AAAI 2022, https://doi.org/10.1609/aaai.v36i5.20512

[41] R. Indhumathi and S. Sathiya Devi. 2022. Healthcare Cramér Generative Adversarial Network (HCGAN). Distrib Parallel Databases 40, 4 (December 2022), 657–673. https://doi.org/10.1007/s10619-021-07346-x

[42] Samer El Kababji, Nicholas Mitsakakis, Xi Fang, Ana-Alicia Beltran-Bless, Greg Pond, Lisa Vandermeer, Dhenuka Radhakrishnan, Lucy Mosquera, Alexander Paterson, Lois Shepherd, Bingshu Chen, William E. Barlow, Julie Gralow, Marie-France Savard, Mark Clemons, and Khaled El Emam. 2023. Evaluating the Utility and Privacy of Synthetic Breast Cancer Clinical Trial Data Sets. JCO Clin Cancer Inform 7 (September 2023). https://doi.org/10.1200/cci.23.00116

[43] Takoua Khorchani, Yojana Gadiya, Gesa Witt, Delia Lanzillotta, Carsten Claussen, and Andrea Zaliani. 2022. SASC: A simple approach to synthetic cohorts for generating longitudinal observational patient cohorts from COVID-19 clinical data. Patterns 3, 4 (April 2022), 100453. https://doi.org/10.1016/j.patter.2022.100453

[44] Martin Klein, Ricardo Moura, and Bimal Sinha. 2021. Multivariate Normal Inference based on Singly Imputed Synthetic Data under Plug-in Sampling. Sankhya B 83, 1 (May 2021), 273–287. https://doi.org/10.1007/s13571-019-00215-9

[45] Naveen Kodali, Jacob Abernethy, James Hays, and Zsolt Kira. 2017. On Convergence and Stability of GANs. arXiv preprint (2017). https://doi.org/10.48550/arXiv.1705.07215

[46] Lisa Kühnel, Julian Schneider, Ines Perrar, Tim Adams, Sobhan Moazemi, Fabian Prasser, Ute Nöthlings, Holger Fröhlich, and Juliane Fluck. 2024. Synthetic data generation for a longitudinal cohort study – evaluation, method extension and reproduction of published data analysis results. Sci Rep 14, 1 (June 2024), 14412. https://doi.org/10.1038/s41598-024-62102-2

[47] Nicholas I.Hsien Kuo, Federico Garcia, Anders Sönnerborg, Michael Böhm, Rolf Kaiser, Maurizio Zazzi, Mark Polizzotto, Louisa Jorm, and Sebastiano Barbieri. 2023. Generating synthetic clinical data that capture class imbalanced distributions with generative adversarial networks: Example using antiretroviral therapy for HIV. J Biomed Inform 144, (August 2023). https://doi.org/10.1016/j.jbi.2023.104436

[48] Nicholas I-Hsien Kuo, Louisa Jorm, and Sebastiano Barbieri. 2023. Synthetic Health-related Longitudinal Data with Mixed-type Variables Generated using Diffusion Models. In NeurIPS 2023 Workshop on Synthetic Data Generation with Generative AI, 2023. Retrieved from https://openreview.net/forum?id=1MV49Ug6q9

[49] Nicholas I-Hsien Kuo, Mark N. Polizzotto, Simon Finfer, Federico Garcia, Anders Sönnerborg, Maurizio Zazzi, Michael Böhm, Rolf Kaiser, Louisa Jorm, and Sebastiano Barbieri. 2022. The Health Gym: synthetic health-related datasets for the development of reinforcement learning algorithms. Sci Data 9, 1 (November 2022), 693. https://doi.org/10.1038/s41597-022-01784-7

[50] Thomas A Lasko. 2014. Efficient Inference of Gaussian-Process-Modulated Renewal Processes with Application to Medical Event Data. In 30th Conference on Uncertainty in Artificial Intelligence, July 23, 2014. AUAI Press, Quebec, 469–476. https://dl.acm.org/doi/10.5555/3020751.3020800

[51] Dongha Lee, Hwanjo Yu, Xiaoqian Jiang, Deevakar Rogith, Meghana Gudala, Mubeen Tejani, Qiuchen Zhang, and Li Xiong. 2020. Generating sequential electronic health records using dual adversarial autoencoder. Journal of the American Medical Informatics Association 27, 9 (2020). https://doi.org/10.1093/jamia/ocaa119

[52] Scott H. Lee. 2018. Natural language generation for electronic health records. NPJ Digit Med 1, 1 (December 2018). https://doi.org/10.1038/s41746-018-0070-0

[53] Ban Li, Senlin Luo, Xiaonan Qin, and Limin Pan. 2021. Improving GAN with inverse cumulative distribution function for tabular data synthesis. Neurocomputing 456, (October 2021), 373–383. https://doi.org/10.1016/j.neucom.2021.05.098

[54] Jin Li, Benjamin J. Cairns, Jingsong Li, and Tingting Zhu. 2023. Generating synthetic mixed-type longitudinal electronic health records for artificial intelligent applications. NPJ Digit Med 6, 1 (May 2023), 98. https://doi.org/10.1038/s41746-023-00834-7

[55] Yi Liu, Jialiang Peng, James J.Q. Yu, and Yi Wu. 2019. PPGAN: Privacy-Preserving Generative Adversarial Network. In 2019 IEEE 25th International Conference on Parallel and Distributed Systems (ICPADS), December 01, 2019. IEEE, 985–989. https://doi.org/10.1109/ICPADS47876.2019.00150

[56] João Lobo, Rui Henriques, and Sara C. Madeira. 2021. G-Tric: generating three-way synthetic datasets with triclustering solutions. BMC Bioinformatics 22, 1 (December 2021), 16. https://doi.org/10.1186/s12859-020-03925-4

[57] Joseph S. Lombardo and Linda J. Moniz. 2008. A method for generation and distribution of synthetic medical record data for evaluation of disease-monitoring systems. Johns Hopkins APL Technical Digest (Applied Physics Laboratory) 27, 4 (2008).

[58] Chang Lu, Chandan K. Reddy, Ping Wang, Dong Nie, and Yue Ning. 2022. Multi-Label Clinical Time-Series Generation via Conditional GAN. arXiv preprint (April 2022). https://doi.org/10.48550/arXiv.2204.04797

[59] José Manuel Martínez-García, Carmen Paz Suárez-Araujo, and Patricio García Báez. 2012. SNEOM: A Sanger Network Based Extended Over-Sampling Method. Application to Imbalanced Biomedical Datasets. In Lecture Notes in Computer Science. 584–592. https://doi.org/10.1007/978-3-642-34478-7_71

[60] Scott McLachlan, Kudakwashe Dube, Thomas Gallagher, Jennifer A. Simmonds, and Norman Fenton. 2019. Realistic Synthetic Data Generation: The ATEN Framework. In Communications in Computer and Information Science. 497–523. https://doi.org/10.1007/978-3-030-29196-9_25

[61] Sandro De Paula Mendonca, Yvan Pereira Dos Santos Brito, Carlos Gustavo Resque Dos Santos, Rodrigo Do Amor Divino Lima, Tiago Davi Oliveira De Araujo, and Bianchi Serique Meiguins. 2020. Synthetic Datasets Generator for Testing Information Visualization and Machine Learning Techniques and Tools. IEEE Access 8, (2020), 82917–82928. https://doi.org/10.1109/ACCESS.2020.2991949

[62] Olof Mogren. 2016. C-RNN-GAN: Continuous recurrent neural networks with adversarial training. arXiv prerint (2016). https://doi.org/10.48550/arXiv.1611.09904

[63] Lucy Mosquera, Khaled El Emam, Lei Ding, Vishal Sharma, Xue Hua Zhang, Samer El Kababji, Chris Carvalho, Brian Hamilton, Dan Palfrey, Linglong Kong, Bei Jiang, and Dean T. Eurich. 2023. A method for generating synthetic longitudinal health data. BMC Med Res Methodol 23, 1 (December 2023). https://doi.org/10.1186/s12874-023-01869-w

[64] Giannis Nikolentzos, Michalis Vazirgiannis, Christos Xypolopoulos, Markus Lingman, and Erik G. Brandt. 2023. Synthetic electronic health records generated with variational graph autoencoders. NPJ Digit Med 6, 1 (December 2023). https://doi.org/10.1038/s41746-023-00822-x

[65] Beata Nowok, Gillian M. Raab, and Chris Dibben. 2016. Synthpop: Bespoke Creation of Synthetic Data in R. J Stat Softw 74, 11 (October 2016). https://doi.org/10.18637/jss.v074.i11

[66] Anna Oganian and Josep Domingo-Ferrer. 2017. Local synthesis for disclosure limitation that satisfies probabilistic k-anonymity criterion. Trans Data Priv 10, 1 (2017), 61–81. https://doi.org/10.5555/3121409.3121412

[67] Mourad Ouzzani, Hossam Hammady, Zbys Fedorowicz, and Ahmed Elmagarmid. 2016. Rayyan—a web and mobile app for systematic reviews. Syst Rev 5, 1 (December 2016), 210. https://doi.org/10.1186/s13643-016-0384-4

[68] Chao Pang, Xinzhuo Jiang, Nishanth Parameshwar Pavinkurve, Krishna S. Kalluri, Elise L. Minto, Jason Patterson, Linying Zhang, George Hripcsak, Gamze Gürsoy, Noémie Elhadad, and Karthik Natarajan. 2024. CEHR-GPT: Generating Electronic Health Records with Chronological Patient Timelines. arXiv preprint (February 2024). https://doi.org/10.48550/arXiv.2402.04400

[69] Yubin Park, Joydeep Ghosh, and Mallikarjun Shankar. 2013. Perturbed Gibbs Samplers for Generating Large-Scale Privacy-Safe Synthetic Health Data. In 2013 IEEE International Conference on Healthcare Informatics, September 2013. IEEE, 493–498. https://doi.org/10.1109/ICHI.2013.76

[70] M. Peréz-Ortiz, P. Tiňo, R. Mantiuk, and C. Hervás-Martínez. 2019. Exploiting Synthetically Generated Data with Semi-Supervised Learning for Small and Imbalanced Datasets. Proceedings of the AAAI Conference on Artificial Intelligence 33, 01 (July 2019), 4715–4722. https://doi.org/10.1609/aaai.v33i01.33014715

[71] R Core Team. 2021. R: A Language and Environment for Statistical Computing. https://doi.org/https://doi.org/10.59350/t79xt-tf203

[72] Gillian M Raab, Beata Nowok, and Chris Dibben. 2018. Practical Data Synthesis for Large Samples. Journal of Privacy and Confidentiality 7, 3 (February 2018), 67–97. https://doi.org/10.29012/jpc.v7i3.407

[73] Alec Radford, Jeffrey Wu, Rewon Child, David Luan, Dario Amodei, and Ilya Sutskever. 2019. Language Models are Unsupervised Multitask Learners. Retrieved from https://cdn.openai.com/better-language-models/language_models_are_unsupervised_multitask_learners.pdf (Aug 2024)

[74] T E Raghunathan, Peter W Solenberger, and John Van Hoewyk. 2002. IVEware: Imputation and Variance Estimation Software User Guide.

[75] Giorgia Ramponi, Pavlos Protopapas, Marco Brambilla, and Ryan Janssen. 2018. T-CGAN: Conditional Generative Adversarial Network for Data Augmentation in Noisy Time Series with Irregular Sampling. arXiv preprint (2018). https://doi.org/10.48550/arXiv.1811.08295

[76] M. Schuster and K.K. Paliwal. 1997. Bidirectional recurrent neural networks. IEEE Transactions on Signal Processing 45, 11 (1997), 2673–2681. https://doi.org/10.1109/78.650093

[77] Stanislau Semeniuta, Aliaksei Severyn, and Erhardt Barth. 2017. A hybrid convolutional variational autoencoder for text generation. In EMNLP 2017 - Conference on Empirical Methods in Natural Language Processing, Proceedings, 2017. https://doi.org/10.18653/v1/d17-1066

[78] Shiven Sharma, Colin Bellinger, Bartosz Krawczyk, Osmar Zaiane, and Nathalie Japkowicz. 2018. Synthetic Oversampling with the Majority Class: A New Perspective on Handling Extreme Imbalance. In 2018 IEEE International Conference on Data Mining (ICDM), November 2018. IEEE, 447–456. https://doi.org/10.1109/ICDM.2018.00060

[79] Jingpu Shi, Dong Wang, Gino Tesei, and Beau Norgeot. 2022. Generating high-fidelity privacy-conscious synthetic patient data for causal effect estimation with multiple treatments. Front Artif Intell 5, (September 2022). https://doi.org/10.3389/frai.2022.918813

[80] Meemansa Sood, Akrishta Sahay, Reagon Karki, Mohammad Asif Emon, Henri Vrooman, Martin Hofmann-Apitius, and Holger Fröhlich. 2020. Realistic simulation of virtual multi-scale, multi-modal patient trajectories using Bayesian networks and sparse auto-encoders. Sci Rep 10, 1 (July 2020), 10971. https://doi.org/10.1038/s41598-020-67398-4

[81] Meemansa Sood, Ulrike Suenkel, Anna-Katharina von Thaler, Helena U. Zacharias, Kathrin Brockmann, Gerhard W. Eschweiler, Walter Maetzler, Daniela Berg, Holger Fröhlich, and Sebastian Heinzel. 2023. Bayesian network modeling of risk and prodromal markers of Parkinson's disease. PLoS One 18, 2 (February 2023), e0280609. https://doi.org/10.1371/journal.pone.0280609

[82] Paola Stolfi, Ilaria Valentini, Maria Concetta Palumbo, Paolo Tieri, Andrea Grignolio, and Filippo Castiglione. 2020. Potential predictors of type-2 diabetes risk: machine learning, synthetic data and wearable health devices. BMC Bioinformatics 21, S17 (December 2020), 508. https://doi.org/10.1186/s12859-020-03763-4

[83] Hongda Sun, Hongzhan Lin, and Rui Yan. 2023. Collaborative Synthesis of Patient Records through Multi-Visit Health State Inference. arXiv preprint (December 2023). https://doi.org/10.48550/arXiv.2312.14646

[84] Bo Tang and Haibo He. 2015. KernelADASYN: Kernel based adaptive synthetic data generation for imbalanced learning. In 2015 IEEE Congress on Evolutionary Computation (CEC), May 2015. IEEE, 664–671. https://doi.org/10.1109/CEC.2015.7256954

[85] Brandon Theodorou, Shrusti Jain, Cao Xiao, and Jimeng Sun. 2024. ConSequence: Synthesizing Logically Constrained Sequences for Electronic Health Record Generation. Proceedings of the AAAI Conference on Artificial Intelligence 38, 14 (March 2024), 15355–15363. https://doi.org/10.1609/aaai.v38i14.29460

[86] Brandon Theodorou, Cao Xiao, and Jimeng Sun. 2023. Synthesize high-dimensional longitudinal electronic health records via hierarchical autoregressive language model. Nat Commun 14, 1 (August 2023), 5305. https://doi.org/10.1038/s41467-023-41093-0

[87] Amirsina Torfi and Edward A. Fox. 2020. CorGAN: Correlation-Capturing Convolutional Generative Adversarial Networks for Generating Synthetic Healthcare Records. The International FLAIRS Conference Proceedings 33, 0 (January 2020). https://doi.org/10.32473/flairs.v33i0

[88] Amirsina Torfi, Edward A. Fox, and Chandan K. Reddy. 2022. Differentially private synthetic medical data generation using convolutional GANs. Inf Sci (N Y) 586, (2022). https://doi.org/10.1016/j.ins.2021.12.018

[89] Manhar Walia, Brendan Tierney, and Susan Mckeever. 2020. Synthesising Tabular Data using Wasserstein Conditional GANs with Gradient Penalty (WCGAN-GP). In Proceedings of The 28th Irish Conference on Artificial Intelligence and Cognitive Science, December 2020. Technological University Dublin, Dublin, 325–336. https://doi.org/https://doi.org/10.21427/E6WA-SZ92

[90] Jason Walonoski, Mark Kramer, Joseph Nichols, Andre Quina, Chris Moesel, Dylan Hall, Carlton Duffett, Kudakwashe Dube, Thomas Gallagher, and Scott McLachlan. 2018. Synthea: An approach, method, and software mechanism for generating synthetic patients and the synthetic electronic health care record. Journal of the American Medical Informatics Association 25, 3 (March 2018), 230–238. https://doi.org/10.1093/jamia/ocx079

[91] Zhiqiang Wan, Yazhou Zhang, and Haibo He. 2017. Variational autoencoder based synthetic data generation for imbalanced learning. In 2017 IEEE Symposium Series on Computational Intelligence (SSCI), November 2017. IEEE, 1–7. https://doi.org/10.1109/SSCI.2017.8285168

[92] Lu Wang, Wei Zhang, and Xiaofeng He. 2019. Continuous Patient-Centric Sequence Generation via Sequentially Coupled Adversarial Learning. In Lecture Notes in Computer Science. 36–52. https://doi.org/10.1007/978-3-030-18579-4_3

[93] Xiaoxia Wang, Yifei Lin, Yun Xiong, Suhua Zhang, Yanming He, Yuqing He, Zhikun Zhang, Joseph M. Plasek, Li Zhou, David W. Bates, and Chunlei Tang. 2022. Using an optimized generative model to infer the progression of complications in type 2 diabetes patients. BMC Med Inform Decis Mak 22, 1 (December 2022), 174. https://doi.org/10.1186/s12911-022-01915-5

[94] Zifeng Wang and Jimeng Sun. 2022. PromptEHR: Conditional Electronic Healthcare Records Generation with Prompt Learning. In Proceedings of the 2022 Conference on Empirical Methods in Natural Language Processing, 2022. Association for Computational Linguistics, Stroudsburg, PA, USA, 2873–2885. https://doi.org/10.18653/v1/2022.emnlp-main.185

[95] Philipp Wendland, Colin Birkenbihl, Marc Gomez-Freixa, Meemansa Sood, Maik Kschischo, and Holger Fröhlich. 2022. Generation of realistic synthetic data using Multimodal Neural Ordinary Differential Equations. NPJ Digit Med 5, 1 (August 2022), 122. https://doi.org/10.1038/s41746-022-00666-x

[96] Jingyi Xu, Zilu Zhang, Tal Friedman, Yitao Liang, and Guy Van Den Broeck. 2018. A semantic loss function for deep learning with symbolic knowledge. In 35th

International Conference on Machine Learning, ICML 2018. Retrieved from https://proceedings.mlr.press/v80/xu18h

[97] Lei Xu, Maria Skoularidou, Alfredo Cuesta-Infante, and Kalyan Veeramachaneni. 2019. Modeling tabular data using conditional GAN. In Advances in Neural Information Processing Systems, 2019. https://dl.acm.org/doi/10.5555/3454287.3454946

[98] Jinsung Yoon, Lydia N. Drumright, and Mihaela van der Schaar. 2020. Anonymization Through Data Synthesis Using Generative Adversarial Networks (ADS-GAN). IEEE J Biomed Health Inform 24, 8 (August 2020), 2378–2388. https://doi.org/10.1109/JBHI.2020.2980262

[99] Jinsung Yoon, Daniel Jarrett, and Mihaela Van Der Schaar. 2019. Time-series Generative Adversarial Networks. In 33rd Conference on Neural Information Processing Systems, 2019. https://dl.acm.org/doi/10.5555/3454287.3454781

[100] Jinsung Yoon, Michel Mizrahi, Nahid Farhady Ghalaty, Thomas Jarvinen, Ashwin S. Ravi, Peter Brune, Fanyu Kong, Dave Anderson, George Lee, Arie Meir, Farhana Bandukwala, Elli Kanal, Sercan Arık, and Tomas Pfister. 2023. EHR-Safe: generating high-fidelity and privacy-preserving synthetic electronic health records. NPJ Digit Med 6, 1 (December 2023). https://doi.org/10.1038/s41746-023-00888-7

[101] Lantao Yu, Weinan Zhang, Jun Wang, and Yong Yu. 2017. SeqGAN: Sequence generative adversarial nets with policy gradient. In 31st AAAI Conference on Artificial Intelligence, AAAI 2017, 2017. https://doi.org/10.1609/aaai.v31i1.10804

[102] Mandi Yu, Yulei He, and Trivellore E. Raghunathan. 2022. A Semiparametric Multiple Imputation Approach to Fully Synthetic Data for Complex Surveys. J Surv Stat Methodol 10, 3 (June 2022), 618–641. https://doi.org/10.1093/jssam/smac016

[103] Mojtaba Zare and Janusz Wojtusiak. 2018. Weighted Itemsets Error (WIE) Approach for Evaluating Generated Synthetic Patient Data. In 2018 17th IEEE International Conference on Machine Learning and Applications (ICMLA), December 2018. IEEE, 1017–1022. https://doi.org/10.1109/ICMLA.2018.00166

[104] Jun Zhang, Graham Cormode, Cecilia M. Procopiuc, Divesh Srivastava, and Xiaokui Xiao. 2017. PrivBayes. ACM Transactions on Database Systems 42, 4 (December 2017), 1–41. https://doi.org/10.1145/3134428

[105] Ziqi Zhang, Chao Yan, Thomas A Lasko, Jimeng Sun, and Bradley A Malin. 2021. SynTEG: a framework for temporal structured electronic health data simulation. Journal of the American Medical Informatics Association 28, 3 (March 2021), 596–604. https://doi.org/10.1093/jamia/ocaa262

[106] Ziqi Zhang, Chao Yan, and Bradley A. Malin. 2022. Keeping synthetic patients on track: feedback mechanisms to mitigate performance drift in longitudinal health data simulation. J Am Med Inform Assoc 29, 11 (October 2022), 1890–1898. https://doi.org/10.1093/jamia/ocac131

[107] Ziqi Zhang, Chao Yan, Diego A. Mesa, Jimeng Sun, and Bradley A. Malin. 2020. Ensuring electronic medical record simulation through better training, modeling, and evaluation. Journal of the American Medical Informatics Association 27, 1 (2020). https://doi.org/10.1093/jamia/ocz161
